# Dynamic Model and Inverse Kinematic Identification of a 3-DOF Manipulator Using RLSPSO

**DOI:** 10.3390/s20020416

**Published:** 2020-01-11

**Authors:** Josias Batista, Darielson Souza, Laurinda dos Reis, Antônio Barbosa, Rui Araújo

**Affiliations:** 1 Robotics, Automation and Control Research Group (GPAR), Federal University of Ceará, Fortaleza-CE 60455-760, Brazil; darielson@dee.ufc.br (D.S.); laurinda@dee.ufc.br (L.d.R.); 2 Federal Institute of Ceará–IFCE Campus Maracanaú, Maracanaú-CE 61925-315, Brazil; barbosa@dee.ufc.br; 3 Institute of Systems and Robotics (ISR-UC), University of Coimbra, Pólo II, PT-3030-290 Coimbra, Portugal; rui@isr.uc.pt; 4 Department of Electrical and Computer Engineering (DEEC-UC), University of Coimbra, Pólo II, PT-3030-290 Coimbra, Portugal

**Keywords:** least Squares, recursive least squares, inverse kinematics, dynamic model, improved RLS with PSO

## Abstract

This paper presents the identification of the inverse kinematics of a cylindrical manipulator using identification techniques of Least Squares (LS), Recursive Least Square (RLS), and a dynamic parameter identification algorithm based on Particle Swarm Optimization (PSO) with search space defined by RLS (RLSPSO). A helical trajectory in the cartesian space is used as input. The dynamic model is found through the Lagrange equation and the motion equations, which are used to calculate the torque values of each joint. The torques are calculated from the values of the inverse kinematics, identified by each algorithm and from the manipulator joint speeds and accelerations. The results obtained for the trajectories, speeds, accelerations, and torques of each joint are compared for each algorithm. The computational costs as well as the Multi-Correlation Coefficient ($R^{2}$) are computed. The results demonstrated that the identification accuracy of RLSPSO is better than that of LS and PSO. This paper brings an improvement in RLS because it is a method with high complexity, so the proposed method (hybrid) aims to improve the computational cost and the results of the classic RLS.

## 1. Introduction

The diffusion of several systems in industrial environments has led over the years to the fact that several identification methods were developed to monitor and control various models of plants such as mobile robots or manipulator robots giving them the ability to operate accurately and efficiency [1]. These robots must perform tasks with great perfection and safety. For this purpose, they need appropriate kinematic and dynamic models that represent the real manipulator.

Nonlinearity and time variation are characteristics of some systems and to model and control them one often wants to use linear models. One of the difficulties of some processes is when operating conditions change thus giving a valuable choice of model partitions during the upgrade. Some methodologies of estimation of model parameters were proposed as the recursive least squares method (RLS). According to the work presented in [2] the RLS method updates a vector of parameters and has a lower computational cost than the non-recursive least squares method. The work of Hafezi et al. [3] addresses two recursive identification methods with ARMA noise with applications in identification of bilinear systems were proposed the generalized extended least squares (GELS) and recursive maximum likelihood (RML) methods.

The quality of data obtained by the system can significantly influence the identification of a manipulator model. Generally some data may have poor quality thus interfering the identification process. Its include insufficient input excitation and low signal to noise ratio. Moreover, brief knowledge of the system model can help in the implementation of the control project. Physical systems may include nonlinear and stochastic behaviors and present data outliers. These systems can interferes in the performance of identification algorithms. The Robust Algorithms Approach has relevance when it comes to systems with outliers according to [4]. The appearance of outliers may also compromise the performance of techniques when there is insertion of Gaussian distribution noise in the data samples as presented in [5].

In [6], the LS is used to solve the problem of four degrees of freedom ship manoeuvring. It is used to perform identification modelling with the full-scale trial data. A new transformed multi-innovation least squares (TMILS) algorithm it was used. Ma, J. et al. in [7] proposes a new approach for identifying a Wiener-based model in which the system can be interpreted by an exogenous autoregressive model coupled with least squares and a support vector machine (LSSVM). The parameters were select by adaptive particle swarm optimization (APSO) that obtain better performance in relation of classical PSO.

The work of [8] presents an identification of dynamic parameters of the lower extremity exoskeleton using the Particle Swarm Optimization (PSO) metaheuristic in the search space defined by Recursive Least Square (RLS), thus making it a hybrid method. During the definition in the PSO search space, the hybrid method not only avoids the convergence of parental identification to the local minimum, but also has very accurate results. Particle Swarm Optimization (PSO)-based identification methods with some variations have been shown in [9]. The identification method is applied to a robotic manipulator where the estimated gaps are used to predict joint torques.

The prototype of a 5-DOF hybrid manipulator was developed in research conducted by [10]. In this research the mechanical structure, the kinematics, the dynamics and the control system were presented. In the results the kinematics and dynamics simulations of this manipulator are presented and tests of accuracy and repeatability of the manipulator path and position. In paper [11] to solve the problem of inverse kinematics (IK), reinforcement learning (RL) was used, designed to balance the lower body of a humanoid 3D robot that has 12 degrees of freedom (DOF). The lower body trajectories are learned by RL which are IK solutions that are converted into positions for NAO robot joints. This reduces the learning dimension because RL-integrated IK eliminates the need to use whole humanoid robot (HR) states. The purpose of the work presented in [12] was to create an ABB IRB120 industrial robot representation for simulating and analyzing dynamics and kinematics of the industrial robots by using MapleSim. In addition the paper presents how linear and nonlinear models of the robot can be obtained and makes available them to public.

Dynamic modeling and kinematics analysis of parallel robot was presented in [13]. In research of [14] its refer to inverse kinematics and a new method to identify the parameters of the dynamic model of the manipulator that was the identification of dynamic parameters based on Particle Swarm Optimization (PSO). The dynamic model taking into account the friction of the manipulator joints is determined and the dynamic parameters are defined as a linear form of the identified parameter. PSO is used to minimize the optimum manipulator trajectory parameters.

In [15] used the torque exerted by each joint when performing periodic excited Fourier trajectories. The parameters were divided into a linear and nonlinear part and used the least square linear parameter estimation (LLS) and the double swarm-based particle swarm optimization (DPso) to calculate the linear and nonlinear parts, respectively. The configurations used were simpler and can identify the dynamic parameters, the friction coefficients of the joints. Already in the paper of [16] techniques were used to identify the dynamic parameters in an industrial manipulator robot with 5 degrees of freedom. The parameters were identified using LS, Adaline artificial neural networks, Hopfield artificial neural networks and the extended Kalman Filter. To solve manipulator robots identification problems [17] presented an intelligent approach with PSO that was called the elitist learning strategy (ELS) and the proportional-integral-derivative controller (PID) hybridized approach (ELPIDSO). The parameter identification of robots manipulator was performed to evaluate the performance of the approach. The ELPIDSO was superior to the LS method, genetic algorithm (GA) and SPSO in the estimation of the parameters of the robot manipulators kinetic models.

Based on the review done above this paper aims to identify the inverse kinematics of a cylindrical manipulator using LS, RLS, and RLS with PSO (RLSPSO). The positions of each manipulator joint obtained using the inverse kinematics model calculated from a trajectory in the Cartesian space are used as input data. The model of the manipulator dynamics is calculated using Lagrangean mechanics and the equations of torques of each manipulator joint are presented. The results show the trajectories, speeds, accelerations, and torques of each joint, real and estimated. The computational cost of each algorithm used in the identification as well as the Multi-Correlation Coefficient ($R^{2}$) of each manipulator joint is presented. A discussion of the results is carried out and the advantages and disadvantages of each method are presented. The inverse kinematics identification can be used to generate real-time manipulator trajectories and to generate collision-free trajectories in static and dynamic obstacle environments as well as being used as an approximation of the inverse kinematics model.

### Contributions

This paper improves the results of classic RLS in relation to computational cost of the proposed method. It is used a particle swarm algorithm with an objective function resulting from the RLS covariance matrix. Each method will be presented a quantitative analysis of the results in order to verify the issue of reducing the complexity of calculating the covariance matrices of the algorithms.

The system to be identified is a three phase induction motor driven cylindrical robotic manipulator. The importance of the proposal is due to the issue of different points of operations when the manipulator is driven. The proposed method still works with non-Gassian disturbances in the system inputs, testing its robustness.

This paper is organized as follows. Section 2 presents the characteristics of the manipulator. Section 3 presents the models of direct kinematics and inverse kinematics, the Jacobian model and the dynamic model. Section 4 presents the formulations on how the LS, RLS, and RLSPSO algorithms were used. Section 5 presents the results of the implemented algorithms and the discussions about the work. Finally Section 6 presents the discussions and conclusions are mentioned in Section 7.

## 2. Cylindrical Manipulator

**Figure 1:**
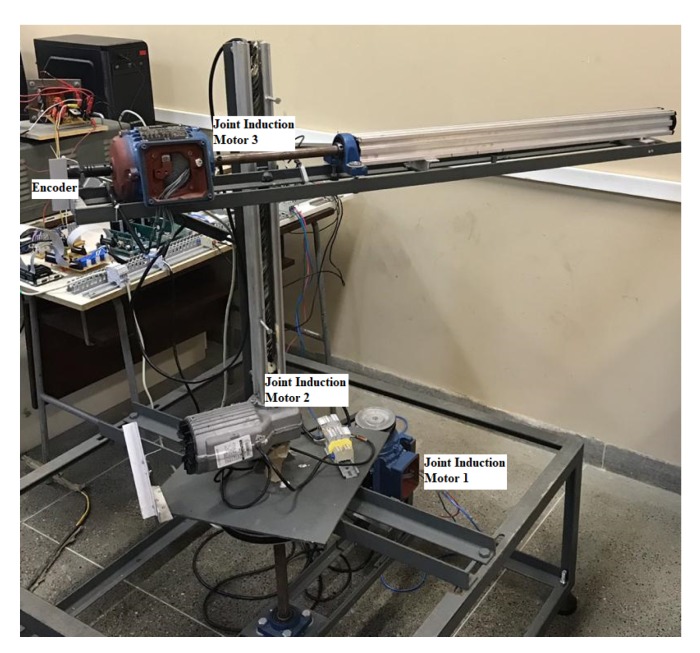
Setup of cylindrical manipulator.

This section presents the characteristics of the manipulator used in this research, as well as the kinematic and dynamic modelling. The kinematics will be modelled using Denavit–Hartenberg (DH) notation, and the dynamic model is based using the Lagrangean Mechanics (LM) modelling method formulations. A cylindrical robotic manipulator that is driven by three phase induction motors was used in this work. As can be seen in Figure 1 the first joint moves around the main axis of the structure (rotational motion), the second and third joints have linear (prismatic) movements, which defines as a RPP (Rotational-Prismatic-Prismatic).

The three-phase induction motors used are of the squirrel cage type. The power of the motors was chosen so that it was possible to move each joint of the manipulator.

### Characteristics of the Manipulator

**Figure 2:**
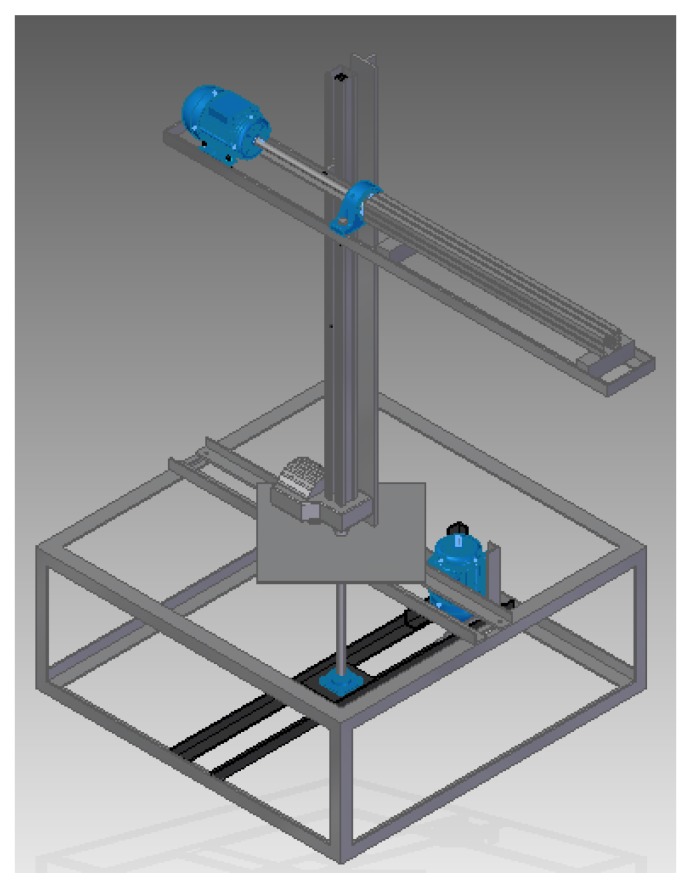
Structure of the cylindrical manipulator—Software Solid Edge©.

This subsection presents some physical characteristics of the manipulator under study. To calculate the torques of each joint it was necessary to find the masses of the robot and the simplest form was through a modelling software Solid Edge© that was able to provide this information [18]. In Figure 2 presents the computational modelling of the manipulator.

**Figure 3:**
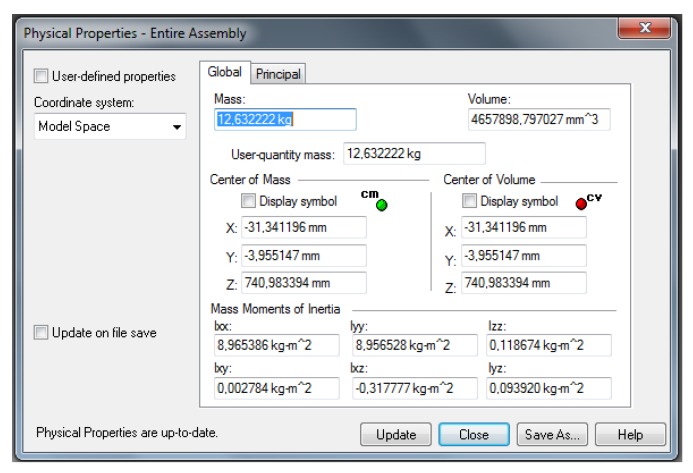
Physical properties of the manipulator joint 2—Software Solid Edge©.

Through the software of computer modelling were found the main physical properties of the manipulator as the dimensions, masses and moments of inertia of each joint. Figure 3 shows a modelling software screen with the physical properties of the manipulator only for joint 2.

The values of the masses (*m*) and lengths (*l*) of each link of the manipulator are shown in Table 1.

The information presented in Table 1 will be used for calculating the joints torques 1, 2 and 3.

## 3. Kinematic and Dynamic Modelling of the Robotic Manipulator

The kinematics exposes the relative motion of the reference systems, as the structure moves by relating reference systems to the various portions of the structure [19,20].

### 3.1. Forward Kinematics

**Figure 4:**
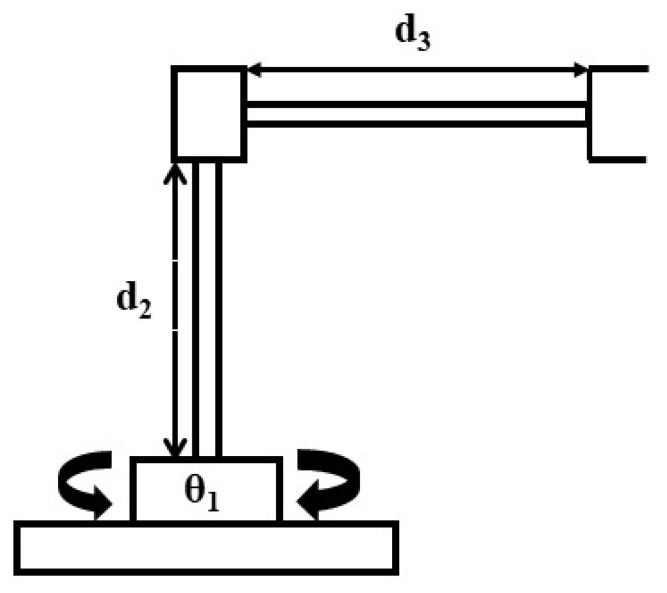
Cylindrical manipulator.

The cylindrical manipulator used in this paper is shown in Figure 4 (Kinematic model of an RPP robotic arm). The kinematic configuration of the manipulator according to the Denavit–Hartenberg (D–H) convention in [21] is established and presented in Table 2. The DH parameters are: twist angles $\alpha_{i}$, link lengths $a_{i}$, joint displacements $q_{i}$, and link offsets $d_{i}$, where i=1,…,n. Obviously, the manipulator has a revolute degree-of-freedom (DOF), and two prismatic movements, it an RPP robotic manipulator.

Using the DH conversion to the parameters shown in Table 2 are the corresponding matrices *A* and *T* [22] given by:

(1) $$A_{1}=\left[\begin{array}{cccc} cos(\theta_{1}) & -sin(\theta_{1}) & 0 & 0\\ sin(\theta_{1}) & cos(\theta_{1}) & 0 & 0\\ 0 & 0 & 1 & 0.245\\ 0 & 0 & 0 & 1 \end{array}\right],$$

(2) $$A_{2}=\left[\begin{array}{cccc} 1 & 0 & 0 & 0\\ 0 & 0 & 1 & 0\\ 0 & -1 & 0 & d_{2}\\ 0 & 0 & 0 & 1 \end{array}\right],$$

(3) $$A_{3}=\left[\begin{array}{cccc} 1 & 0 & 0 & 0\\ 0 & 1 & 0 & 0\\ 0 & 0 & 1 & d_{3}\\ 0 & 0 & 0 & 1 \end{array}\right],$$

(4) $$T_{3}^{0}=A_{1}A_{2}A_{3}=\left[\begin{array}{cccc} cos(\theta_{1}) & 0 & -sin(\theta_{1}) & -sin\left(\theta_{1}\right)\left(d_{3}+0.35\right)\\ sin(\theta_{1}) & 0 & cos(\theta_{1}) & cos\left(\theta_{1}\right)\left(d_{3}+0.35\right)\\ 0 & -1 & 0 & 0.245+d_{2}\\ 0 & 0 & 0 & 1 \end{array}\right].$$

Any final position (end-effector) of the manipulator can be found in the Cartesian space from the coordinates in the joint space, as noted in Equation (5):

(5) $$\left[\begin{array}{c} P_{x}\\ P_{y}\\ P_{z} \end{array}\right]=\left[\begin{array}{c} -sin\left(\theta_{1}\right)\left(d_{3}+0.35\right)\\ cos\left(\theta_{1}\right)\left(d_{3}+0.35\right)\\ 0.245+d_{2} \end{array}\right]$$

### 3.2. Inverse Kinematics

Inverse kinematics defines the configuration, the values of the joint variables, that the manipulator must have for the position and orientation of a chosen point. One method to solve the problem of inverse kinematics of a manipulator is by the geometric method [22]. Applying this method it can be found that $\theta_{1}$ is defined by:

(6) $$\theta_{1}=Atan2\left(P_{x},P_{y}\right)$$

The parameter of link 2 is prismatic, as can be seen in Figure 1, and $d_{2}$ is in the same axis $z_{1}$ given by:

(7) $$d_{2}=P_{z}-0.245$$

In the case of the third parameter $d_{3}$, it will move in the plane formed by *x* and *y* and can be determined by:

(8) $$d_{3}=\left(\sqrt{P_{x}^{2}+P_{y}^{2}}\right)-0.35$$

Equations (6)–(8) are the solutions for the cylindrical manipulator inverse kinematics problem and will be used to perform position control and manipulator path and trajectory generation.

### 3.3. Jacobian

The relationship between the Cartesian (end-effector) and joint speeds of a manipulator is given by the Jacobian [22]. As can be seen in Figure 4 a cylindrical manipulator has the following variables of the joints, $q=(\theta_{1},\;d_{2},\;d_{3})$.

Since the manipulator has a revolute joint and two prismatic joints, i.e., three joints, the Jacobian matrix in this case is of dimensions 6×3 and is of the form:

(9) $$J\left(q\right)=\left[\begin{array}{ccc} z_{0}\times \left(o_{3}-o_{0}\right) & z_{1} & z_{2}\\ z_{0} & o_{0} & o_{0} \end{array}\right]$$

where we have $z_{o}={\left[0\;0\;1\right]}^{T}=z_{1}$ and $o_{0}={\left[0\;0\;0\right]}^{T}$; $z_{2}$ an $o_{3}$ are given by:

(10) $$z_{2}=\left[\begin{array}{c} sen(\theta_{1})\\ cos(\theta_{1})\\ 0 \end{array}\right]$$

(11) $$o_{3}=\left[\begin{array}{c} -cos\left(\theta_{1}\right)\left(l_{3}+0.35\right)\\ -sen\left(\theta_{1}\right)\left(l_{3}+0.35\right)\\ 0 \end{array}\right]$$

Substituting each matrix and performing the necessary operations, considering $d_{3}=l_{3}+0.35$, we have the Jacobian matrix 6×3, given by:

(12) $$\left[\begin{array}{c} \dot{x}\\ \dot{y}\\ \dot{z}\\ \omega_{x}\\ \omega_{y}\\ \omega_{z} \end{array}\right]=\left[\begin{array}{ccc} -cos\left(\theta_{1}\right)d_{3} & 0 & sen(\theta_{1})\\ -sen\left(\theta_{1}\right)d_{3} & 0 & cos(\theta_{1})\\ 0 & 1 & 0\\ 0 & 0 & 0\\ 0 & 0 & 0\\ 1 & 0 & 0 \end{array}\right].\left[\begin{array}{c} {\dot{\theta }}_{1}\\ {\dot{d}}_{2}\\ {\dot{d}}_{3} \end{array}\right]$$

This reveals that it is impossible to perform a rotation around the $x_{0}$ and $y_{0}$ axes. The Jacobian in relation to the linear speed of the end-effector can be obtained considering only the first three matrix lines that is

(13) $$J=\left[\begin{array}{ccc} -cos\left(\theta_{1}\right)\left(d_{3}+0.35\right) & 0 & sen(\theta_{1})\\ -sen\left(\theta_{1}\right)\left(d_{3}+0.35\right) & 0 & cos(\theta_{1})\\ 0 & 1 & 0 \end{array}\right]$$

### 3.4. Dynamic Modelling

The dynamics of the manipulator displays the between the position-speed-acceleration-torque relationship of the joints. Therefore, the dynamic modelling of an industrial robot aims to know the relationship between the movement of the robot and the forces applied to it [19,23]. The model of the manipulator dynamics can be obtained using the Euler–Lagrange formulation, [24,25]. The model equation is of the form shown below:

(14) $$L\left(q,\dot{q}\right)=K\left(q,\dot{q}\right)-P\left(q\right),$$

where *L* is the Lagrangian; *K* is the kinetic energy and *P* is the potential energy (see Appendix A.1). For the cylindrical manipulator under study was calculated the kinetic and potential energy and then applied the formulation based on the Lagrangian [23].

From the Jacobian Equation (12) one can determine the speeds and the equations of the kinetic energy, after performing some operations and mathematical transformations. Potential energy equations could be obtained from classical mechanics [23]. From the Lagrange Equation (14) the system motion equations given by:

(15) $$\frac{d}{dt}\left[\frac{\partial L}{\partial \dot{q}}\right]-\frac{\partial L}{\partial q}=\tau$$

where $\tau \in {ℜ}^{n}$, are the torques applied to the joints. Thus, considering the kinetic energy of the manipulator the dynamic equation of the manipulator can be written in simplified form as:

(16) $$M\left(q\right)\ddot{q}+C\left(q,\dot{q}\right)\dot{q}+G\left(q\right)=\tau ,$$

where, *q*, $\dot{q}$, $\ddot{q}$ $\in {ℜ}^{n}$ indicate the positions, speeds and accelerations joint’s, respectively; M(q) is the inertial matrix; $C\in {ℜ}^{n}$ is the matrix that describes the centripetal and Coriolis forces and $G\frac{\partial g}{\partial q}$ $\in {ℜ}^{n}$ is the gravity matrix.

Applying the Lagrange formulation Equation (14) and from the equation of motion Equation (15), performing the calculations of the partial derivatives, it can be obtained the torque equations of each joint of the manipulator [22] which are shown below. The first equation of motion describing the torque of joint 1 is:

(17) $$\begin{array}{c} \tau_{1}=-\left[\left(4m_{1}sen\theta_{1}-4m_{2}cos\theta_{1}\right)d_{3}+I_{3}\right]{\ddot{\theta }}_{1}\\ +\left[\left(m_{1}+m_{2}\right)\left(sen\theta_{1}cos\theta_{1}\right)d_{3}\right]{\ddot{d}}_{3}\\ +\left[\left(m_{1}sen\theta_{1}-m_{2}cos\theta_{1}\right)d_{3}\right]{\dot{\theta }}_{1}^{2}\\ -\left[m_{1}cos\theta_{1}+m_{2}sen\theta_{1}\right]{\dot{d}}_{3}^{2}\\ -\left[\left(m_{1}+m_{2}\right)\left(sen\theta_{1}cos\theta_{1}\right)d_{3}\right]{\dot{\theta }}_{1}{\dot{d}}_{3} \end{array}$$

Likewise by solving the partial derivatives for the second joint of the manipulator we have the torque of the joint 2 found from the equation of motion of the joint 2:

(18) $$\tau_{2}=m_{3}{\ddot{d}}_{2}+g\left(m_{2}+m_{3}\right)$$

Following the same idea the partial derivatives for the third joint of the manipulator, the equation of motion describing the torque of joint 3 is given by:

(19) $$\begin{array}{c} \tau_{3}=\left[m_{1}sen\theta_{1}cos\theta_{1}\right]{\ddot{\theta }}_{1}\\ -\left[2\left(m_{1}sen\theta_{1}+m_{2}cos\theta_{1}\right)\right]{\ddot{d}}_{3}\\ +\left[2d_{3}\left(m_{1}sen\theta_{1}-m_{2}cos\theta_{1}\right)\right]{\dot{\theta }}_{1}^{2}\\ -\left[\left(m_{1}+m_{2}\right)\left(sen\theta_{1}cos\theta_{1}\right)\right]{\dot{\theta }}_{1}{\dot{d}}_{3} \end{array}$$

The terms in the torque equations, ${\ddot{\theta }}_{1},\;{\ddot{d}}_{2},\;{\ddot{d}}_{3}$ are related to the angular accelerations of the links, the terms ${\dot{\theta }}_{1}^{2},\;{\dot{d}}_{2}^{2},\;{\dot{d}}_{3}^{2}$ are the centripetal accelerations, and the terms ${\dot{\theta }}_{1}{\dot{d}}_{2},\;{\dot{\theta }}_{1}{\dot{d}}_{3}$, ${\dot{d}}_{2}{\dot{d}}_{3}$ are the Coriolis accelerations [23].

Equations (17)–(19) were used to calculate the torques of each manipulator joint and the torque values are presented in the following results section.

## 4. Identification Methods

The practice of identification algorithms is interesting for many applications such as supervision, diagnostics, filtering, prediction, signal processing, detection, and variant parameter tracking for adaptive control. In this section, we presente the methods of identification of the inverse kinematics using Least Squares (LS), Recursive Least Squares (RLS), and Recursive Least Squares with Particle Swarm Optimization (RLSPSO) according to the literature [26,27,28].

### 4.1. Least Squares (LS)

The LS method is one of the most well-known and is used in the most diverse areas [29].

Consider the rigid-body dynamics Equation (16). Let us excite it with a control input τ and collect the resulting *q*, $\dot{q}$, and $\ddot{q}$. Assume we have collected θ samples of each element of τ, *q*, $\dot{q}$ and $\ddot{q}$ corresponding to time instants $t_{1},t_{2},\ldots ,t_{k}$, [30]:

(20) $$\Phi \theta =y(q,\dot{q},\ddot{q}),$$

where

$$\Phi =\left[\begin{array}{c} \Phi_{\left[0\right]}\\ \Phi_{\left[1\right]}\\ \vdots \\ \Phi_{\left[n-1\right]} \end{array}\right],\;y=\left[\begin{array}{c} y_{\left[0\right]}\left(q,\dot{q},\ddot{q}\right)\\ y_{\left[1\right]}\left(q,\dot{q},\ddot{q}\right)\\ \vdots \\ y_{\left[n-1\right]}\left(q,\dot{q},\ddot{q}\right) \end{array}\right],$$

and *n* is the total number of sampled data points. The columns of the matrix Φ should be linearly independent for LS to accurately approximate the parameters. The estimation process can be improved using the total proximity of least squares which also considers uncertainties in the regression matrix.

The input τ(t) it is appropriate to stimulate robot dynamics. With this stimulus the vector of identifiable parameters θ can be estimated from the least-squares (LS) sense using some generalized inverse of the information matrix Φ,

(21) $$\hat{\theta }=\Phi^{*}y,$$

where “*” denotes a generalized matrix inverse.

Equation (21) is a solution by the LS method, which is equal to the solution obtained using the pseudo-inverse matrix. This solution will be used to perform the inverse of kinematic identification with LS.

### 4.2. Recursive Least Squares (RLS)

The LS method is based on set of measures and is unsuitable for real-time application. It is necessary to build, update, have available a model of the system during on-line operation [26,28].

A dynamic model taken over a set of data generates constraints that can be presented by a matrix equation which can be written in the regressor form as,

(22) $$y=\phi \hat{\theta }+\xi ,$$

where ϕ is called the matrix of regressors and ξ is the residue. The RLS solution for $\theta_{\left[k\right]}$ takes the following form [31]:

(23) $$\theta_{\left[k\right]}=\theta_{\left[k-1\right]}+L_{\left[k\right]}\left[y_{\left[k\right]}-\phi_{\left[k\right]}^{T}\theta_{\left[k-1\right]}\right],$$

where

(24) $$L_{\left[k\right]}=\frac{P_{\left[k-1\right]}\phi_{\left[k\right]}}{\lambda_{\left[k\right]}+\phi_{\left[k\right]}^{T}P_{\left[k-1\right]}\phi_{\left[k\right]}},$$

and

(25) $$P_{\left[k\right]}=\frac{1}{\lambda_{\left[k\right]}}\left[P_{\left[k-1\right]}-\frac{P_{\left[k-1\right]}\phi_{\left[k\right]}\phi_{\left[k\right]}^{T}P_{\left[k-1\right]}}{\lambda_{\left[k\right]}+\phi_{\left[k\right]}^{T}P_{\left[k-1\right]}\phi_{\left[k\right]}}\right].$$

### 4.3. Recursive Least Squares with Particle Swarm Optimization (RLSPSO)

Particle Swarm Optimization (PSO) is a metaheuristic inspired on social behavior proposed by [32]. The main objective of the algorithm is to search in a given space, through the data permutation of the particles, consequently each particle will be a trajectory in the search space. PSO excels at other algorithms in aspects such as easy implementation and fast convergence. As with other search algorithms, PSO may have particles trapped in local minima locations [33].

The PSO metaheuristic has particles similar to a set of birds that seek the best way to fly taking into account the position and speed of each particle. A convergence curve is used during the execution of the algorithm. Each particle will have its resulting goal depending also on the behaviour of the general population of particles [33]. The position at time *t* is updated by $x_{i}\left(t\right)$ and at future time t+1 will be given in (26).

(26) $$x_{i}\left(t+1\right)=x_{i}\left(t\right)+v_{i}\left(t+1\right),$$

where $v_{i}\left(t\right)$ is the speed [34]. Each particle will present a cognitive component which will be a relation of the distance between itself and the best (optimal solution) besides the social component that is the understanding of the set on the existence of a given particle. For this problem, we used the global PSO (Global best PSO) in which the particle speed is updated by:

(27) $$\begin{array}{c} v_{ij}\left(t+1\right)=v_{ij}\left(t\right)+c_{1}r_{1}\left(t\right)\left[y_{ij}\left(t\right)-x_{ij}\left(t\right)\right]\\ +c_{2}r_{2}\left(t\right)\left[{\hat{y}}_{ij}\left(t\right)-{\hat{x}}_{ij}\left(t\right)\right], \end{array}$$

for $v_{ij}\left(t\right)$ being the speed of the particle, in a given dimension at time *t*. Again, $c_{1}$ and $c_{2}$ are the acceleration parameters. The best particle information is given by ${\hat{y}}_{ij}$ and $y_{ij}$ is the best position from the beginning [34].

Unlike other evolutionary computing techniques in PSO each particle is associated with a speed. Particles fly through the search space with speeds that are dynamically adjusted according to their historical behavior. Finally, the particles have a tendency to traverse the best areas for research to a solution during the search process [34].

For the PSO algorithm (see Algorithm 1) the following values of the elements were used:

- Number of particles = 60 particles;
- Cognitive and social parameters (learning rates): $c_{1}$ = 3.1 and $c_{2}$ = 3.9;
- Iterations = 10 iterations;
- Inertia factor (w) = 1.0;
- Initial population generation = used a rand in a generic equation that is restricted to the interval [0.01, 50].

**Algorithm 1:** PSO Algorithm 1: initiate the swarm of particles and define *P* Matrix; 2: **repeat** 3: **for**
i=1
**to**
*m* 4: **if**
$f\left(x_{i}\right)<f\left(p_{i}\right)$
**then** 5: $p_{i}=x_{i}$; 6: **if**
$f\left(x_{i}\right)<f\left(g\right)$
**then** 7: $g=x_{i}$; 8: **end if** 9: **end if** 10: **for**
j=1
**to**
*n* 11: $r_{1}=rand\left(\right)$, $r_{2}=rand\left(\right)$; 12: $v_{ij}=wv_{ij}+c_{1}r_{1}\left(p_{i}-x_{ij}\right)+c_{2}r_{2}\left(g_{j}-x_{ij}\right)$; 13: **end for** 14: $x_{i}=x_{i}+v_{i}$; 15: **end for** 16: **to** satisfy the stopping criterion 17: Optimal value of *P* convariance matrix

The stopping criterion used in the PSO algorithm was the number of iterations of the algorithm. The PSO metaheuristic has as its mission to minimize the objective function given in Equation (28), with the number of iterations equal to 10, and the algorithm was executed 10 times for obtaining the best result.

(28) $$J_{min}=1-mean\left(R_{J_{i}}^{2}\right),\;i=1,2,3$$

where $R_{J_{i}}^{2}$ is the multiple correlation coefficient applied to joints 1, 2 and 3.

## 5. Results

In this section, we present the results of the identification of the inverse kinematics of the manipulator. Comparisons of actual and estimated values are presented. The results of speeds, accelerations and torques are also presented for each trajectory generated from the identification of LS, RLS, and RLSPSO.

**Figure 5:**
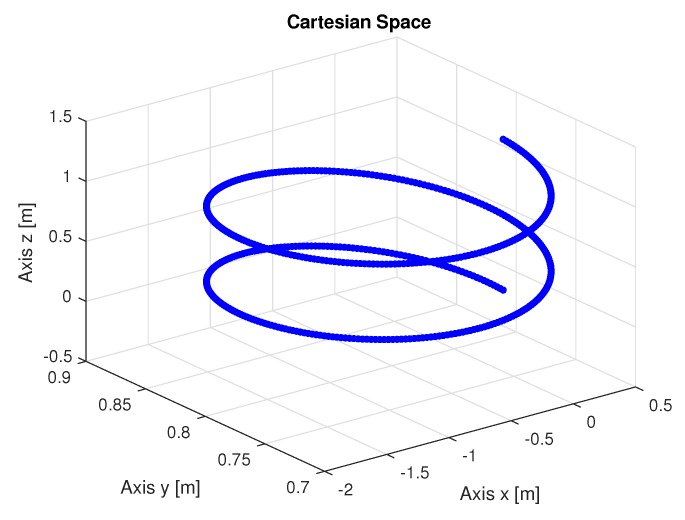
Trajectory executed by the manipulator in Cartesian space.

To perform the manipulator trajectory, an algorithm describing a trajectory shown in Figure 5 was developed where the displacement of each manipulator joint in the cartesian space is performed. This trajectory provides the final manipulator positions collected from the encoders of each manipulator joint that were used as inputs to the algorithms that perform inverse kinematic identification.

In this study, we will consider both noise-free case and noised case in the measurements of position, speed, acceleration. Measurement noises are all considered as the white noise with standard deviation σ=0.05 and the signal to noise ratio is 10 dB.

It is noteworthy that the initial states of the estimation of each algorithm for each joint were initialized with values equal to zero.

### 5.1. Noise-Free results

The present results are data without noise, to make a final analysis. The results of the LS, RLS, and RLSPSO methods will be discussed in this section.

#### 5.1.1. Results of LS

The following are the figures with trajectories, speeds, accelerations and torques of joints 1, 2, and 3 of the manipulator. The trajectories in the space of the joints were obtained from the resolution of the inverse kinematics and the identification using LS using as input the points of the trajectory in the Cartesian space shown in Figure 5.

**Figure 6:**
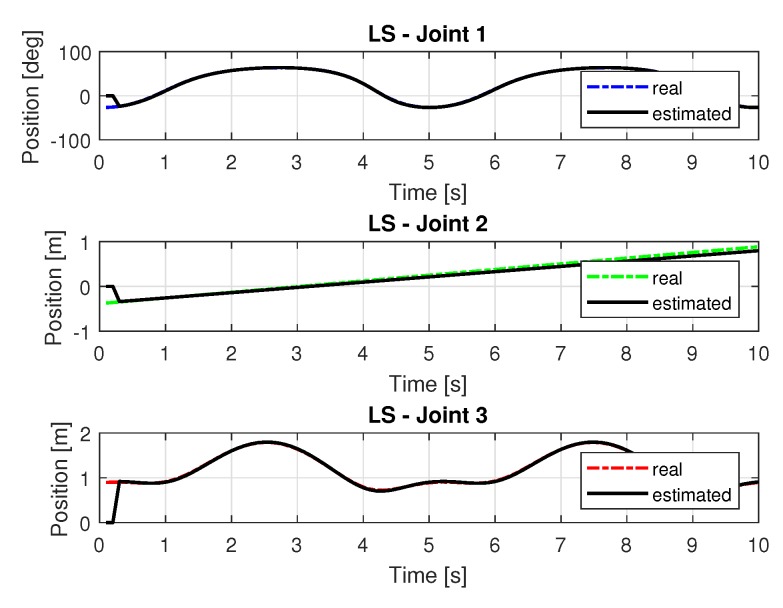
Trajectory in the joint space identified with LS.

Figure 6 shows the trajectories (displacement) of the joint 1, 2, and 3 to perform the path in Cartesian space shown in Figure 5.

**Figure 7:**
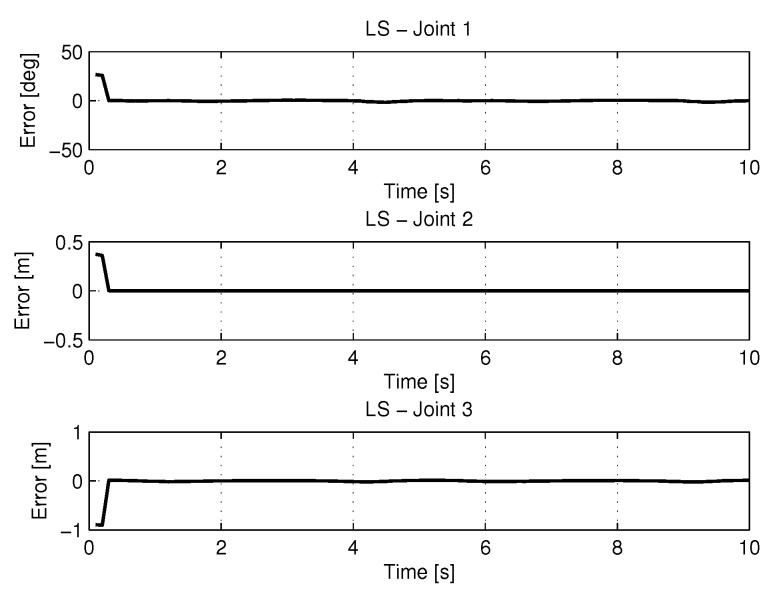
Error of Trajectories identifications with LS.

Figure 7 shows the results of errors in identification the trajectories of each joint using the least squares method.

**Figure 8:**
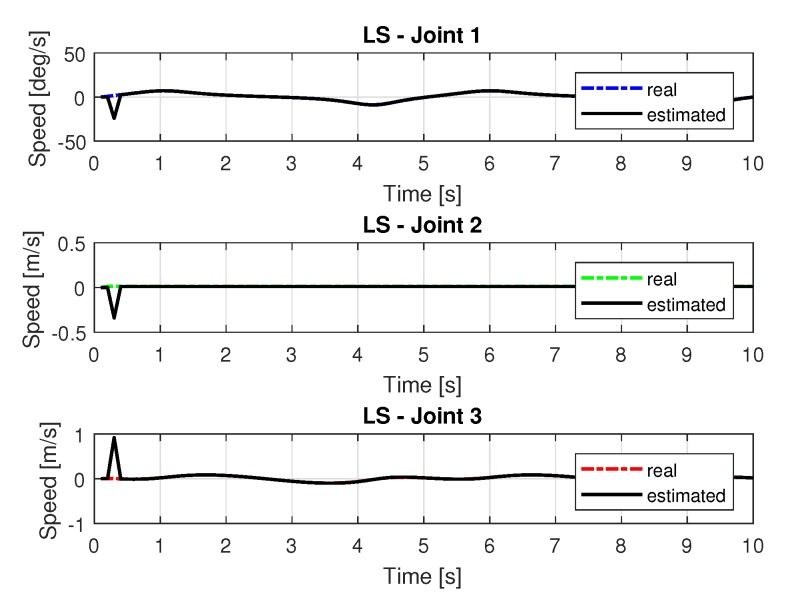
Speed of joints with LS.

**Figure 9:**
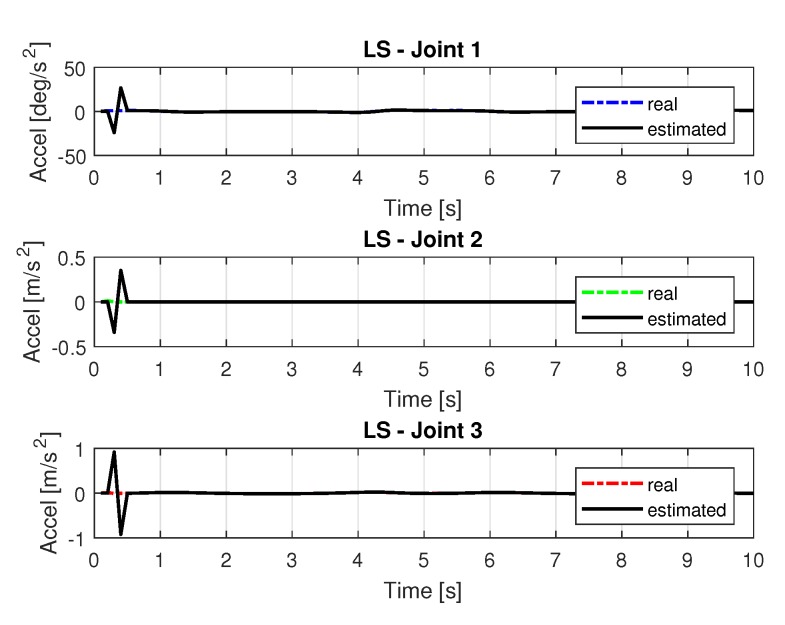
Accelerations of joints with LS.

Figure 8 and Figure 9 shows the speeds and accelerations of joints 1, 2, and 3 to perform the trajectories shown in Figure 6, respectively.

**Figure 10:**
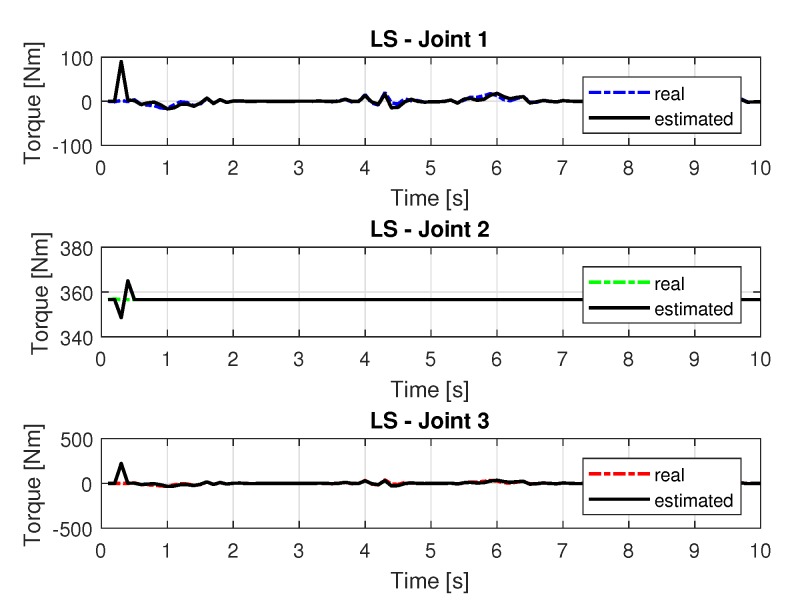
Torque of joints with LS.

The joint torques were obtained from the dynamic model, Equations (17)–(19) of the manipulator and are shown in Figure 10. Torques were calculated by taking the trajectories, speeds and accelerations shown in Figure 6, Figure 8 and Figure 9.

#### 5.1.2. Results of RLS

The following are the paths, speeds, accelerations and torques of joints 1, 2, and 3 of the manipulator. The trajectories in the space of the joints were obtained from the resolution of the inverse kinematics, and the identification using RLS. For this case, we used an exhaustive search to find the best $P_{\left[k\right]}$ weights in order to get better results. The matrix result is:

$$P_{1_{\left[k\right]}}=P_{2_{\left[k\right]}}=P_{3_{\left[k\right]}}=\left[\begin{array}{cccc} 4.999 & 0 & 0 & 0\\ 0 & 4.999 & 0 & 0\\ 0 & 0 & 4.999 & 0\\ 0 & 0 & 0 & 4.999 \end{array}\right]$$

**Figure 11:**
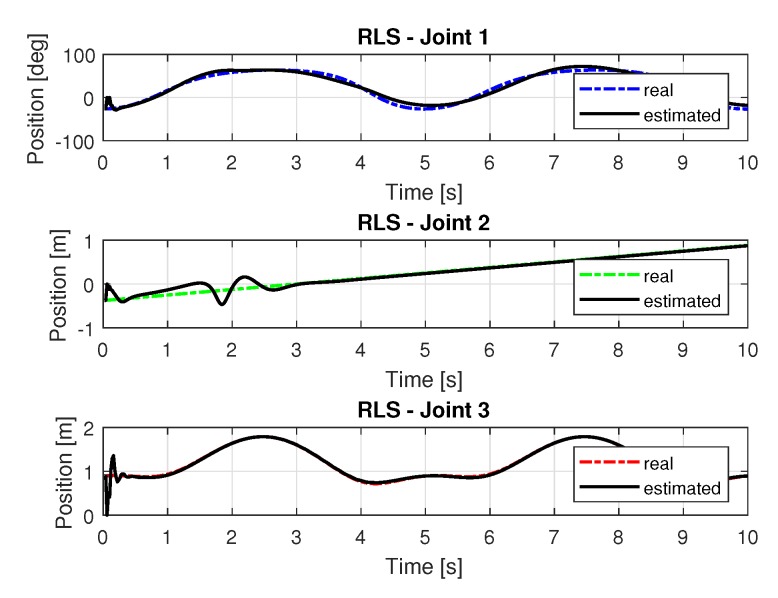
Trajectory in the joint space identified with RLS.

Figure 11 show the trajectories (displacement) of the seals 1, 2, and 3 to perform the path in Cartesian space shown in Figure 5.

**Figure 12:**
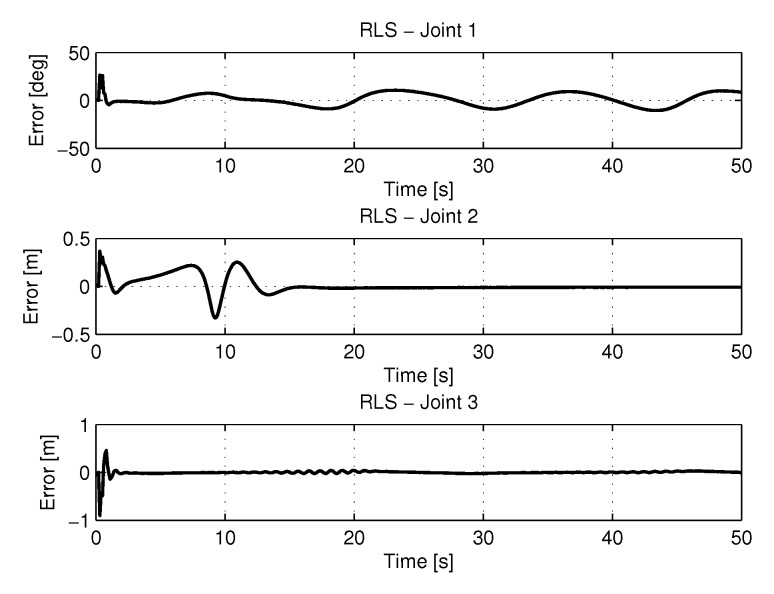
Error of Trajectories identifications with RLS.

Figure 12 shows the results of errors in identification the trajectories of each joint using the recursive least square method.

**Figure 13:**
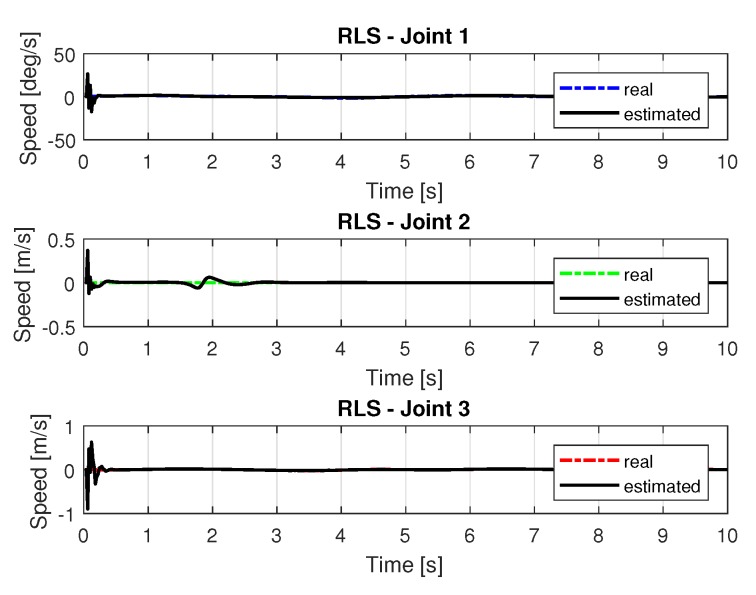
Speed of joints with RLS.

**Figure 14:**
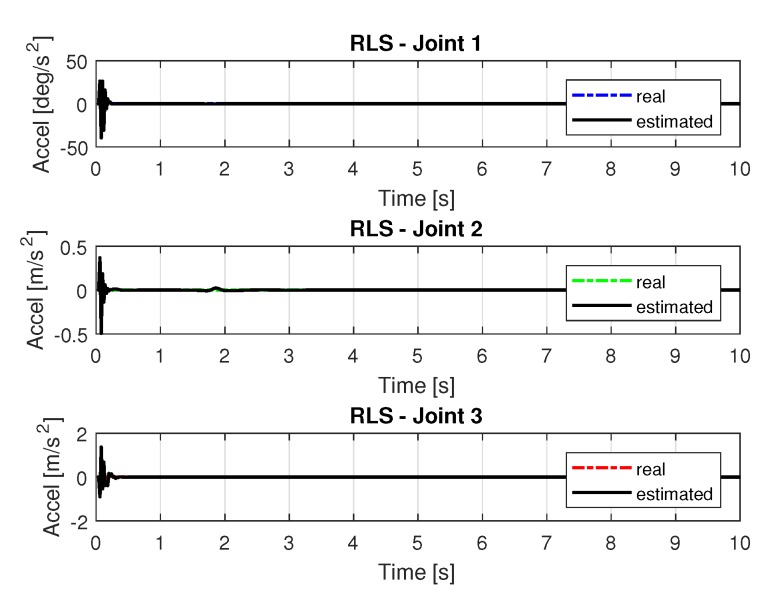
Accelerations of joints with RLS.

Figure 13 and Figure 14 shows the speeds and accelerations of joints 1, 2, and 3 to perform the trajectories shown in Figure 11.

**Figure 15:**
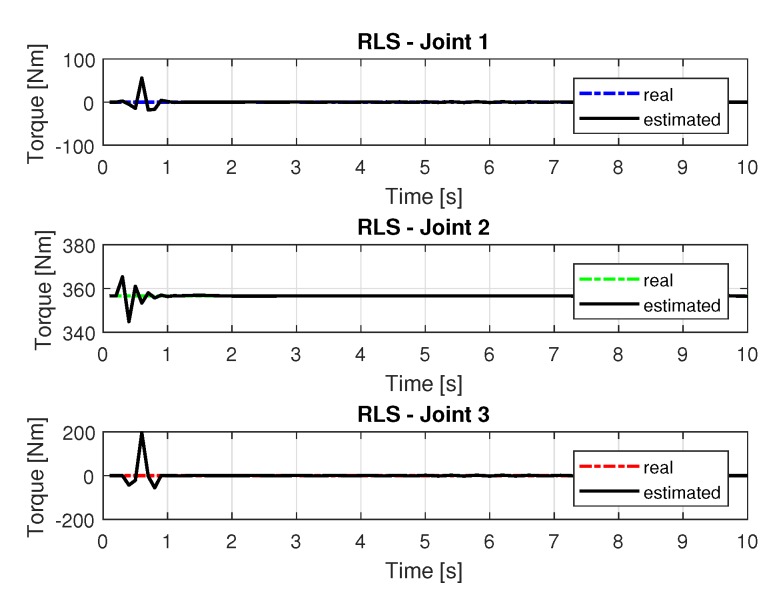
Torque of joints with RLS.

The joint torques were obtained from the dynamic model shown in Equations (17)–(19) of the manipulator presented in Figure 15. Torques were calculated by taking the trajectories, speeds, and accelerations shown in Figure 11, Figure 13 and Figure 14, respectively.

#### 5.1.3. Results of RLSPSO

The following are the paths, speeds, accelerations and torques of joints 1, 2, and 3 of the manipulator. The trajectories in the space of the joints were obtained from the resolution of the inverse kinematics and the identification using RLSPSO. The PSO was used to perform an optimization to find the weights of the matrix $P_{\left[k\right]}$ of the RLS. The matrix $P_{\left[k\right]}$ found by the PSO was

$$P_{1_{\left[k\right]}}=P_{2_{\left[k\right]}}=P_{3_{\left[k\right]}}=\left[\begin{array}{cccc} 10.7521 & 0 & 0 & 0\\ 0 & 39.3081 & 0 & 0\\ 0 & 0 & 26.7325 & 0\\ 0 & 0 & 0 & 36.9065 \end{array}\right]$$

**Figure 16:**
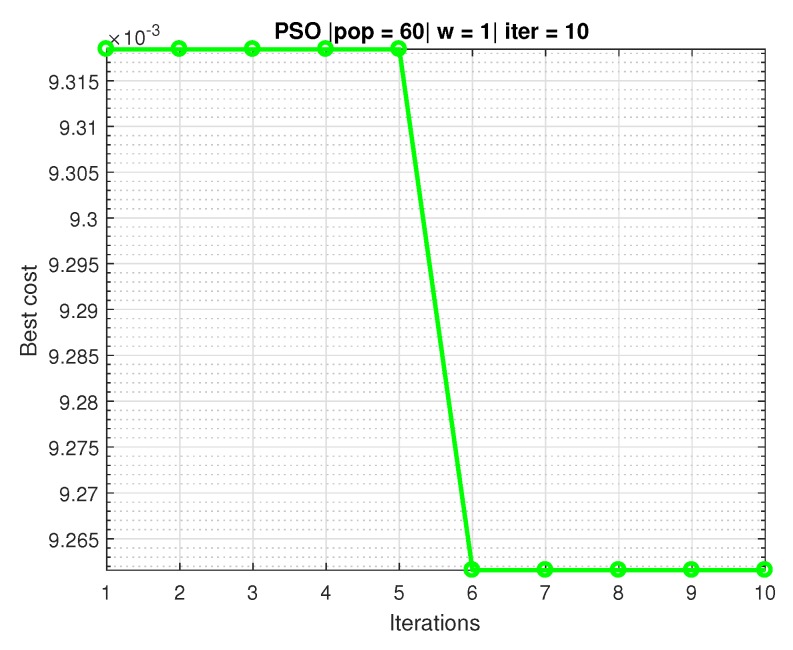
PSO graph converging to 60 particles and 10 iterations.

Figure 16 shows the iterations and the cost in which can be observed that PSO algorithm converges to six iterations with the best cost.

The stopping criterion of the algorithm is number of iterations.

**Figure 17:**
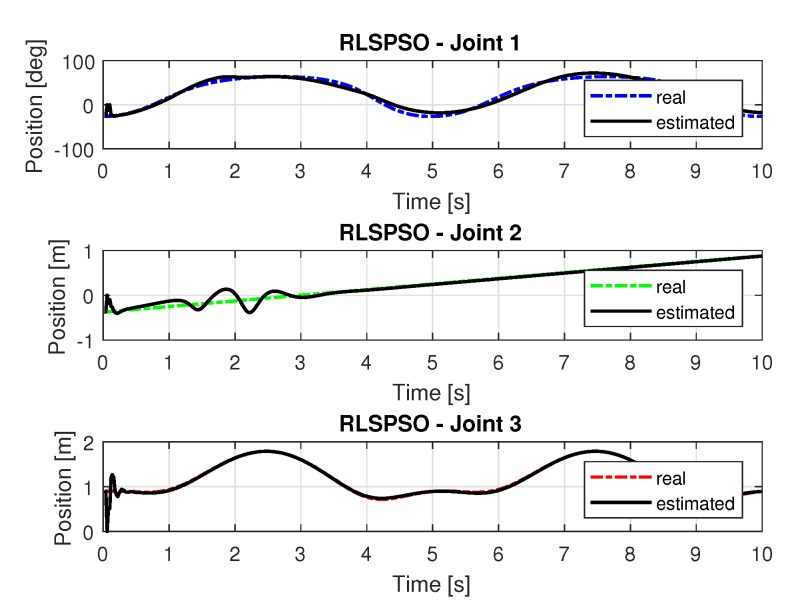
Trajectory in the joint space identified with RLSPSO.

Figure 17 show the trajectories (displacement) of the seals 1, 2, and 3 to perform the path in Cartesian space shown in Figure 5.

**Figure 18:**
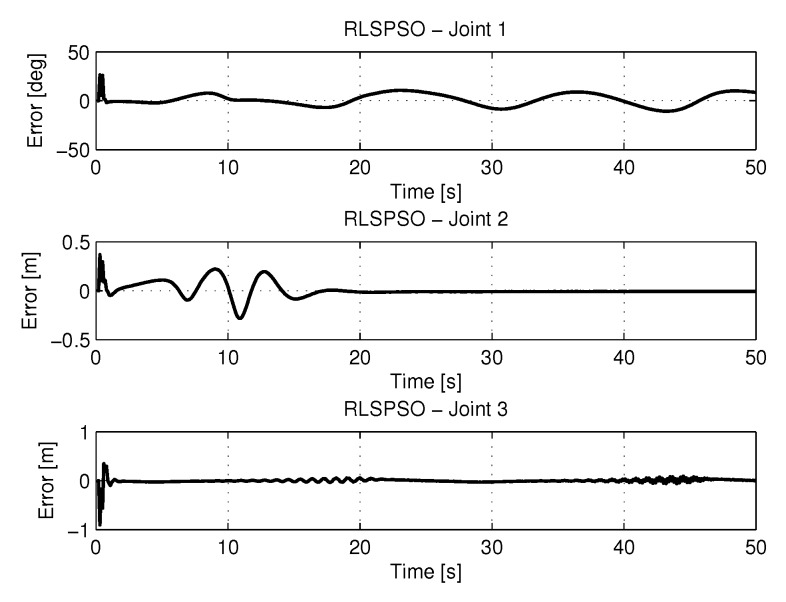
Error of Trajectories identifications with RLSPSO.

Figure 18 shows the results of errors with trajectories identifications using the recursive least square with PSO method.

**Figure 19:**
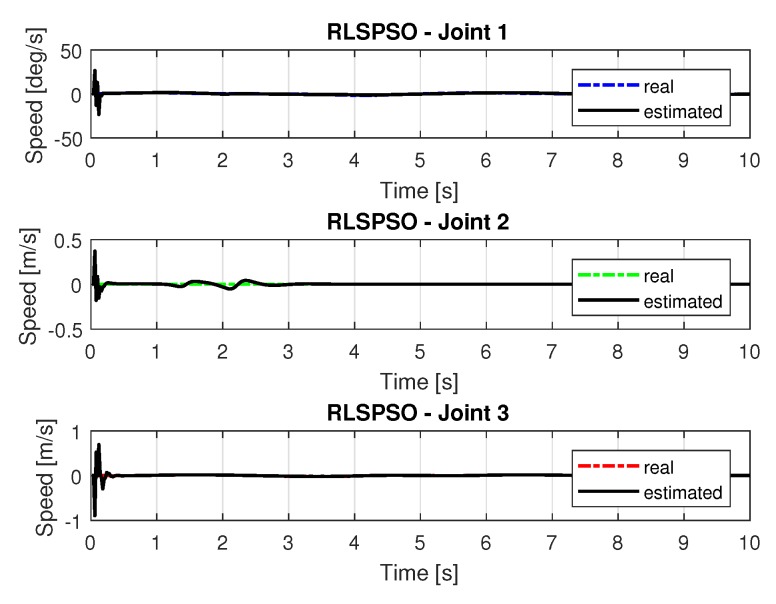
Speed of joints with RLSPSO.

**Figure 20:**
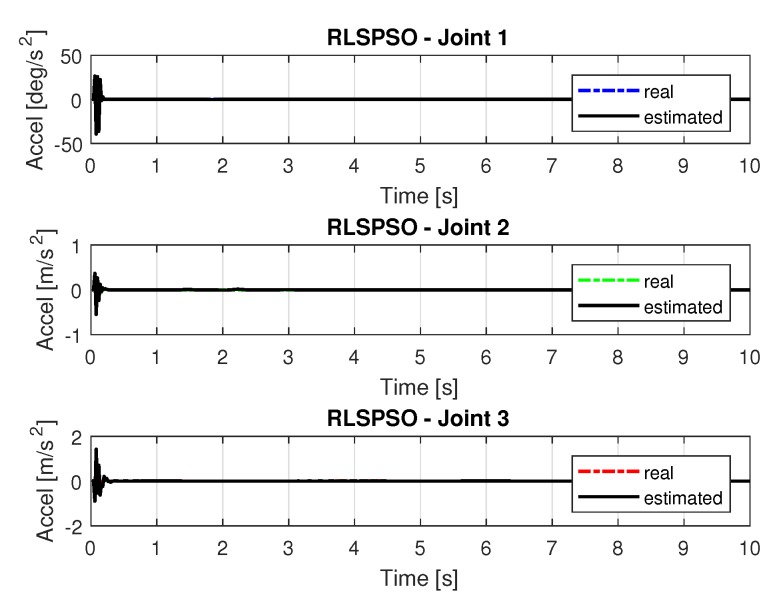
Accelerations of joints with RLSPSO.

Figure 19 and Figure 20 shows the speeds and accelerations of joints 1, 2, and 3 to perform the trajectories shown in Figure 17.

**Figure 21:**
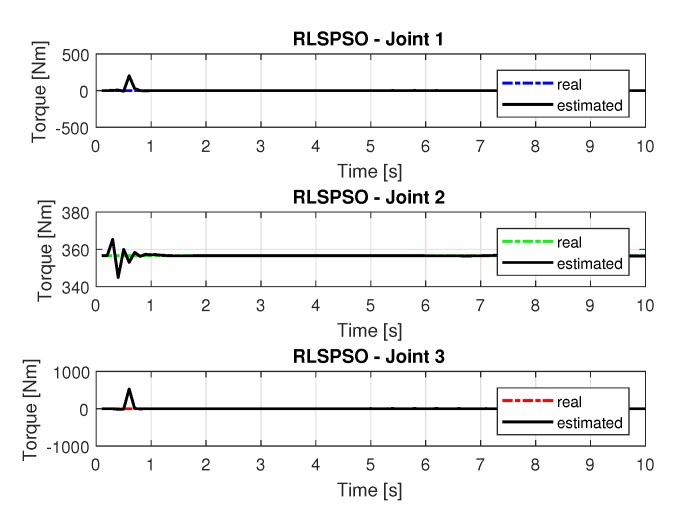
Torque of joints with RLSPSO.

The joint torques were obtained from the dynamic model in Equations (17)–(19) of the manipulator and are shown in Figure 21. Torques were calculated by taking the trajectories, speeds and accelerations shown in Figure 17, Figure 19 and Figure 20.

### 5.2. Results with Noise

Non-Gaussian noise data were used to verify the method identifications with the input data, as well as the appearance of outlies making the estimates difficult.

#### 5.2.1. LS with Noise

**Figure 22:**
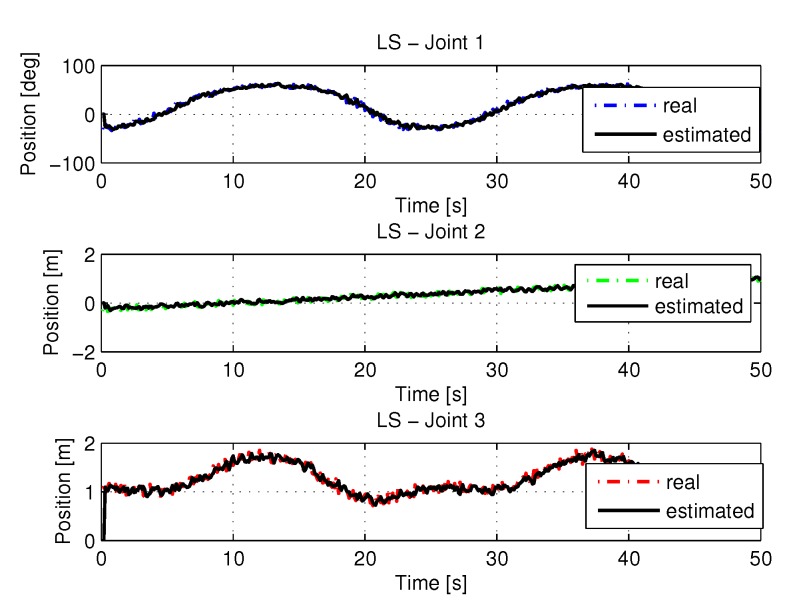
Trajectory in the joint space identified with LS with noise.

The recursive least squares method obtained a high computational effort trying to find the best solution with noisy inputs. Figure 22 show the trajectories (displacement) of the seals 1, 2 and 3 to perform the path in Cartesian space. the speeds and accelerations.

**Figure 23:**
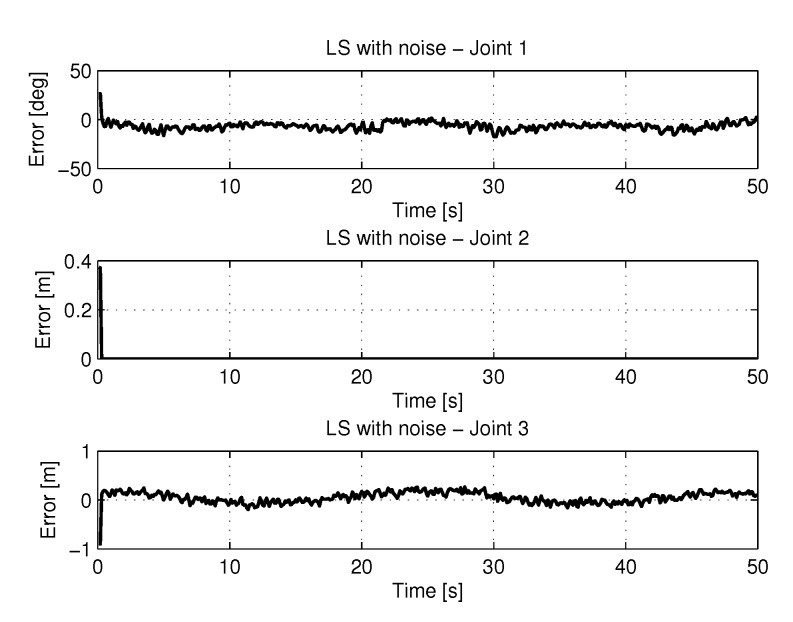
Error of Trajectories identifications with LS with noise.

Figure 23 shows the results of errors in identification the trajectories of each joint using the least square with method with noise.

**Figure 24:**
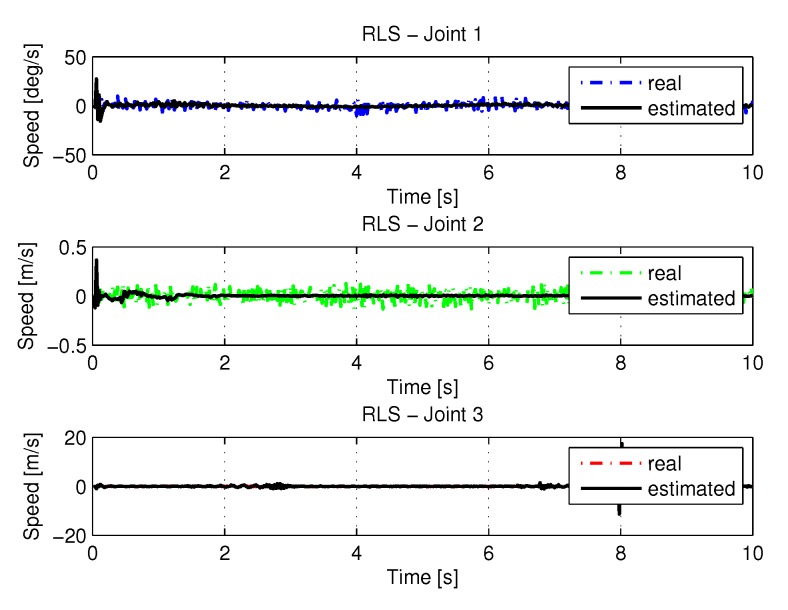
Speed of joints with LS with noise.

**Figure 25:**
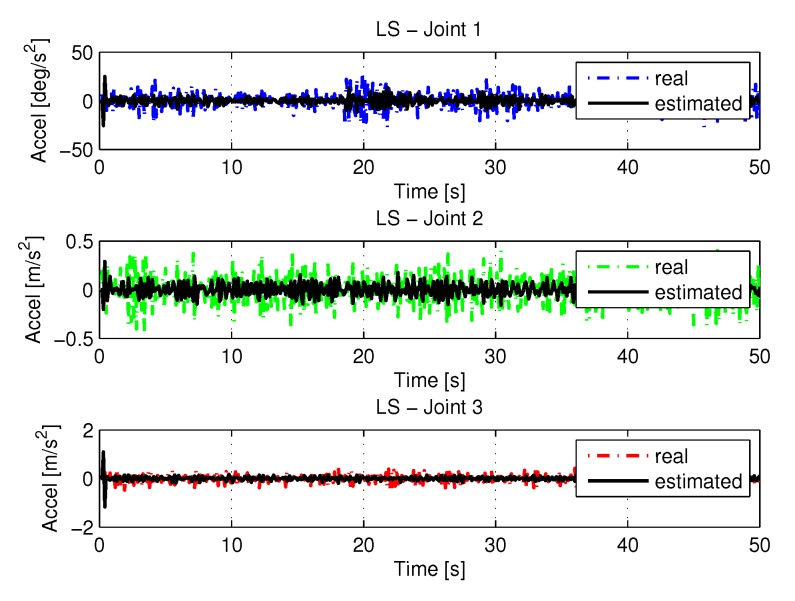
Accelerations of joints with LS with noise.

Figure 24 and Figure 25 shows the the speeds and accelerations of joints 1, 2, and 3 to perform the trajectories shown in Figure 22.

**Figure 26:**
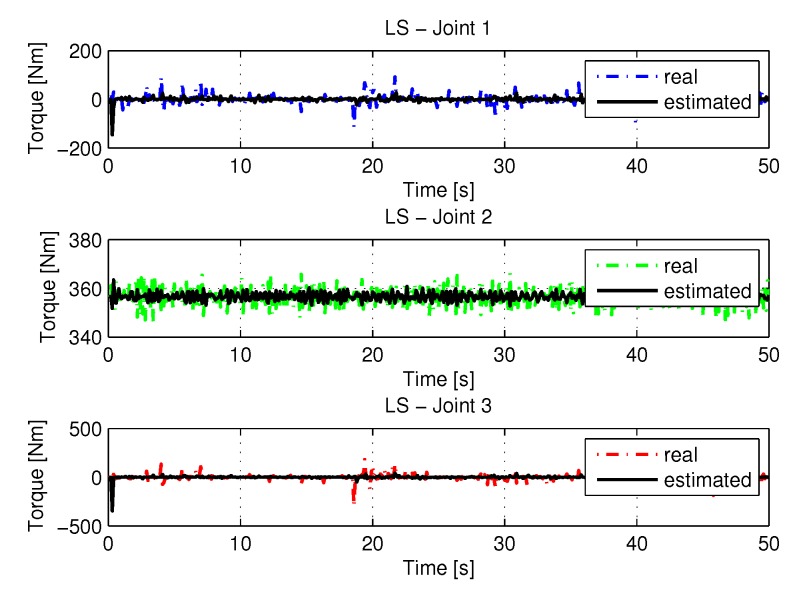
Torque of joints with LS with noise.

Figure 26 shows the results with torque noises using the least square method.

#### 5.2.2. RLS with Noise

The use of noise in the RLS obtained a higher computational effort than no noise where the covariance matrix found was:

$$P_{1_{\left[k\right]}}=P_{2_{\left[k\right]}}=P_{3_{\left[k\right]}}=\left[\begin{array}{cccc} 8.999 & 0 & 0 & 0\\ 0 & 8.999 & 0 & 0\\ 0 & 0 & 8.999 & 0\\ 0 & 0 & 0 & 8.999 \end{array}\right]$$

**Figure 27:**
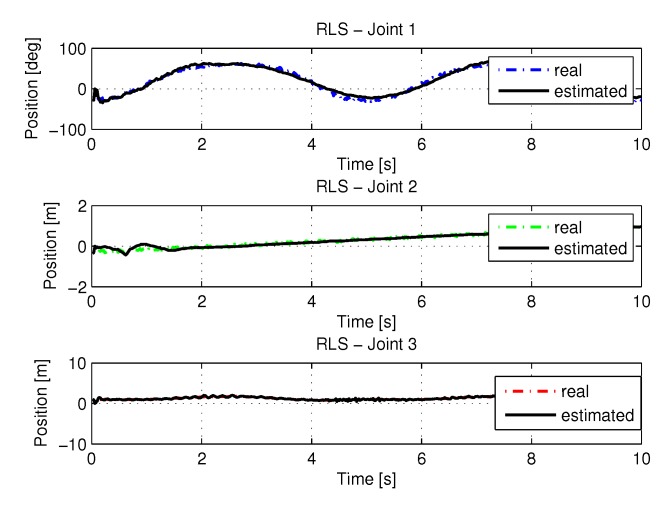
Trajectory in the joint space identified with RLS with noise.

Figure 27 present the results using the noisy RLS method. Input data is the trajectory values shown in the Figure 5.

**Figure 28:**
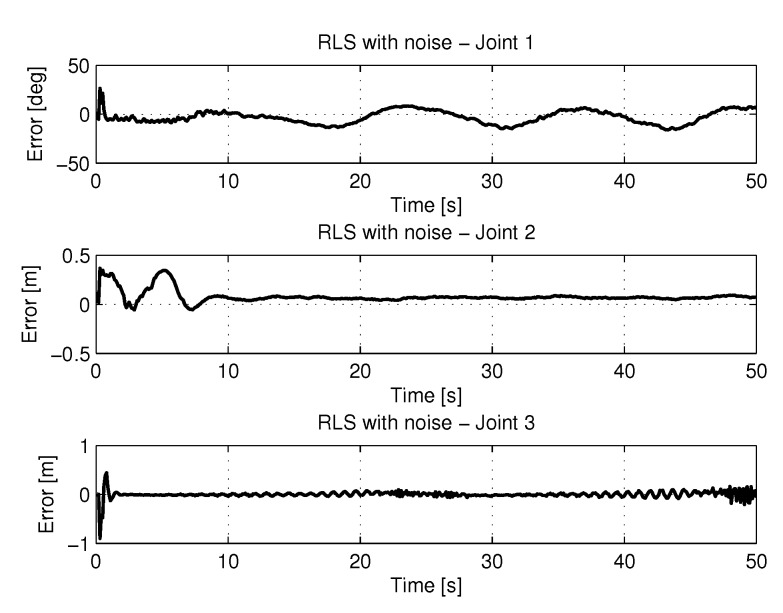
Error of Trajectories identifications with RLS with noise.

Figure 28 shows the results of errors with trajectories identifications using the recursive least square with method with noise.

**Figure 29:**
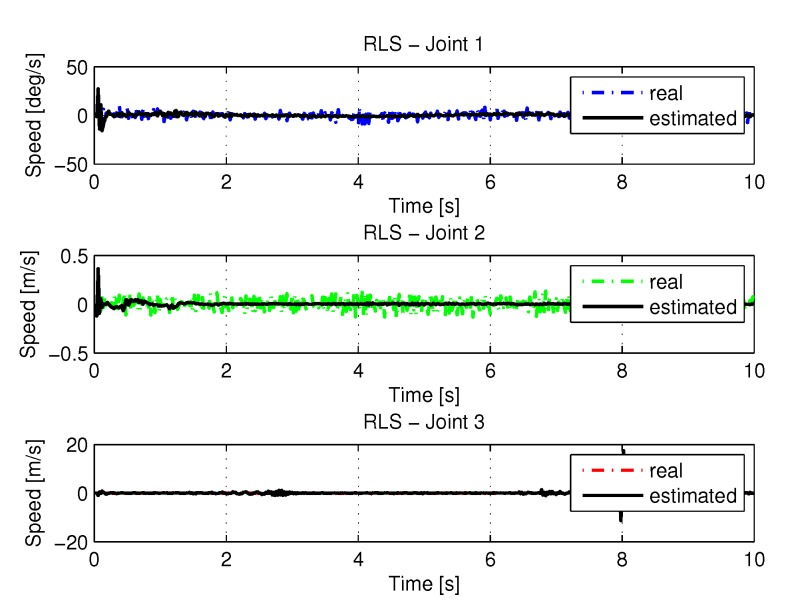
Speed of joints with RLS with noise.

**Figure 30:**
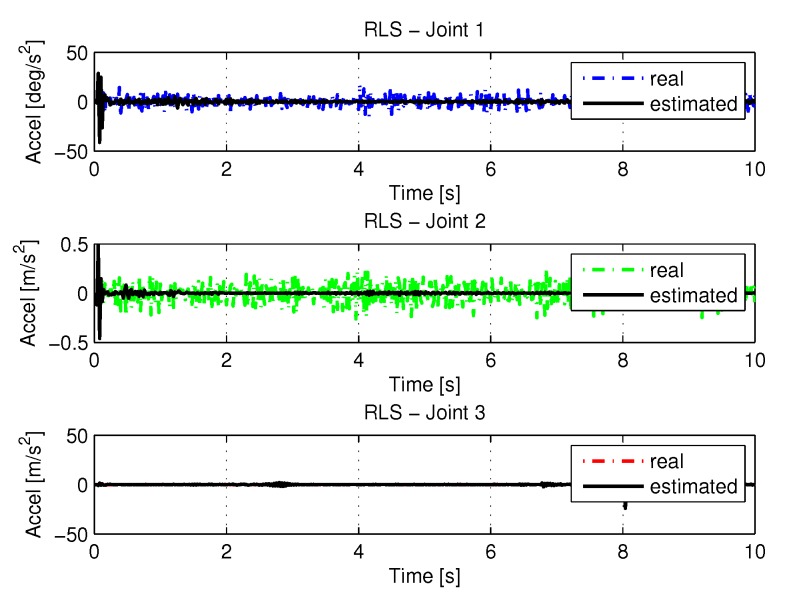
Accelerations of joints with RLS with noise.

Figure 29 and Figure 30 shows the the speeds and accelerations of joints 1, 2, and 3 to perform the trajectories shown in Figure 27.

**Figure 31:**
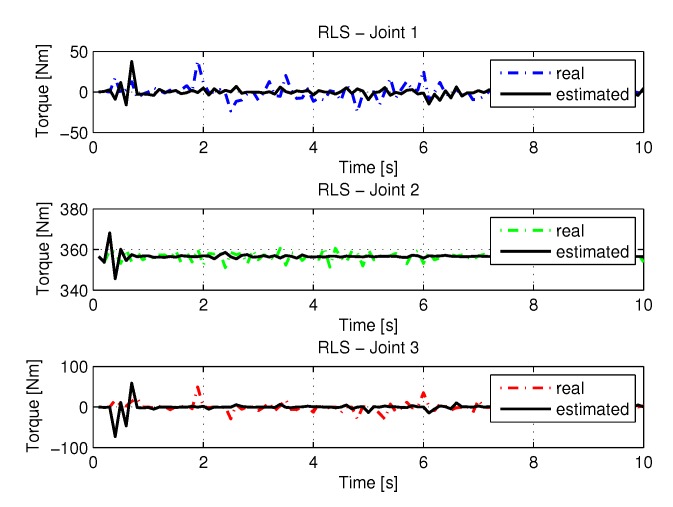
Torque of joints with RLS with noise.

The Figure 31 shows the results with torque noises using the recursive least square method.

#### 5.2.3. RLSPSO with Noise

This section will present the results of the RLSPSO with noise. The matrix $P_{\left[k\right]}$ found by the PSO for RLS with noise was:

$$P_{1_{\left[k\right]}}=P_{2_{\left[k\right]}}=P_{3_{\left[k\right]}}=\left[\begin{array}{cccc} 21.7933 & 0 & 0 & 0\\ 0 & 36.4084 & 0 & 0\\ 0 & 0 & 36.3718 & 0\\ 0 & 0 & 0 & 36.1714 \end{array}\right]$$

**Figure 32:**
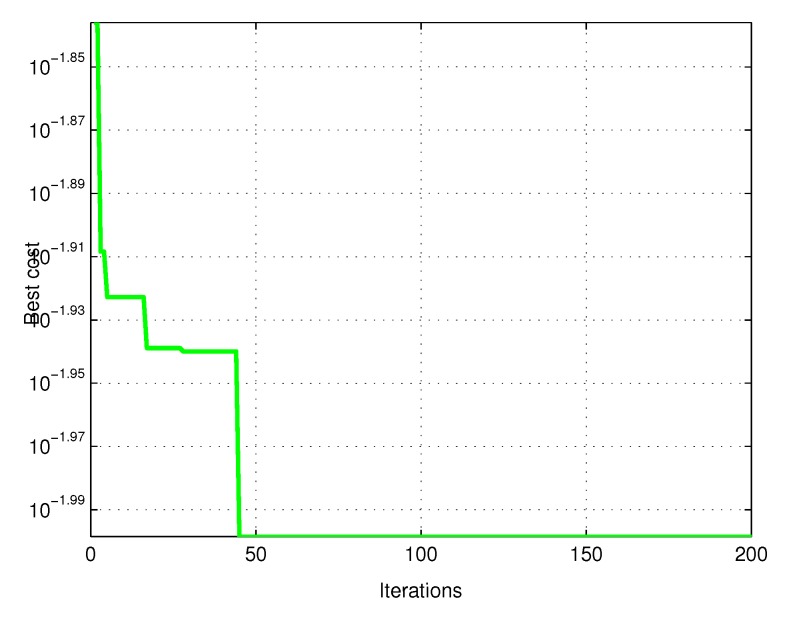
PSO graph converging to 200 particles and 45 iterations.

In Figure 32 shows the iterations and the cost where it can be observed that the PSO algorithm converges to 45 iterations with the best cost. The stopping criterion of the algorithm is number of iterations.

**Figure 33:**
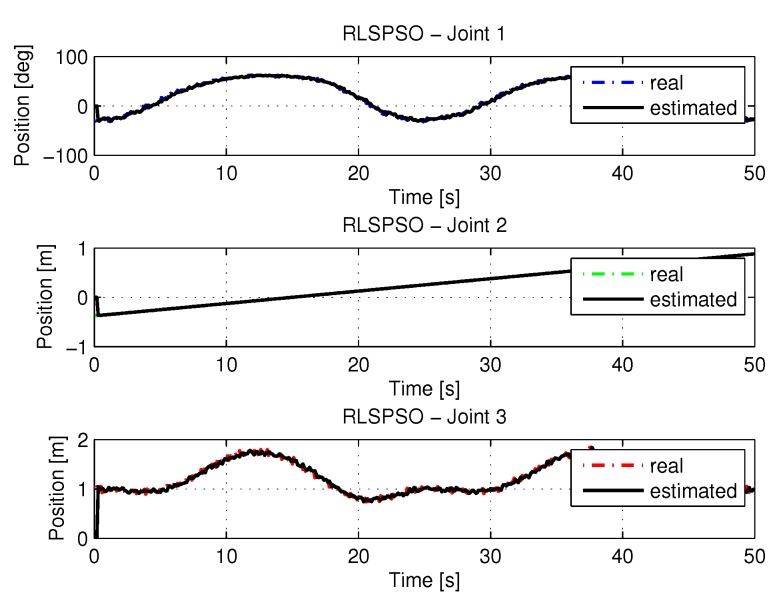
Trajectory in the joint space identified with RLSPSO with noise.

**Figure 34:**
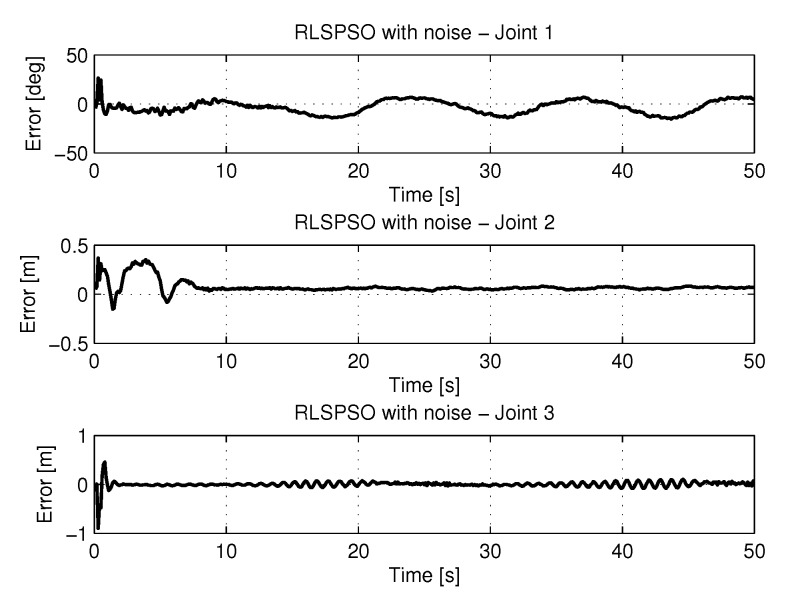
Error of Trajectories identifications with RLSPSO with noise.

Figure 33 show the trajectories (displacement) of the seals 1, 2 and 3 with noisy entries, can be seen below:

Figure 34 shows the results of errors with trajectories identifications using the recursive least square method with PSO with noise.

**Figure 35:**
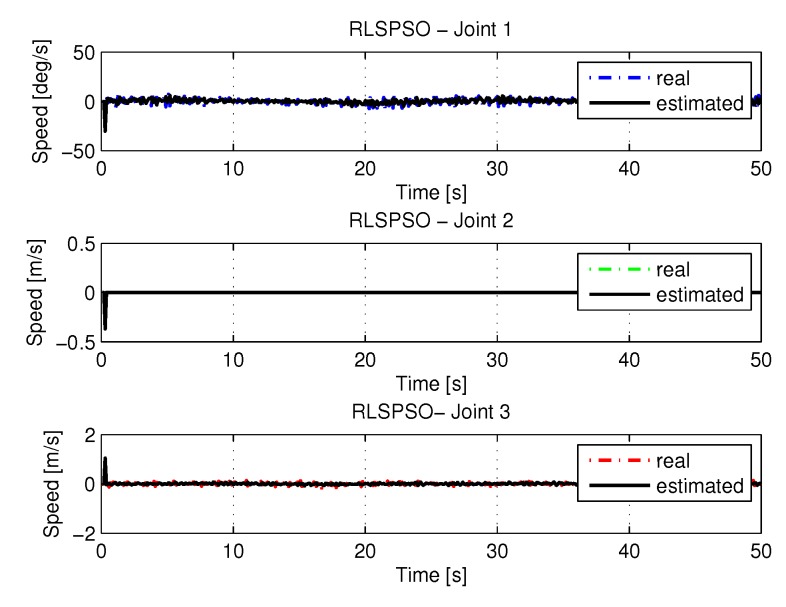
Speed of joints with RLSPSO with noise.

**Figure 36:**
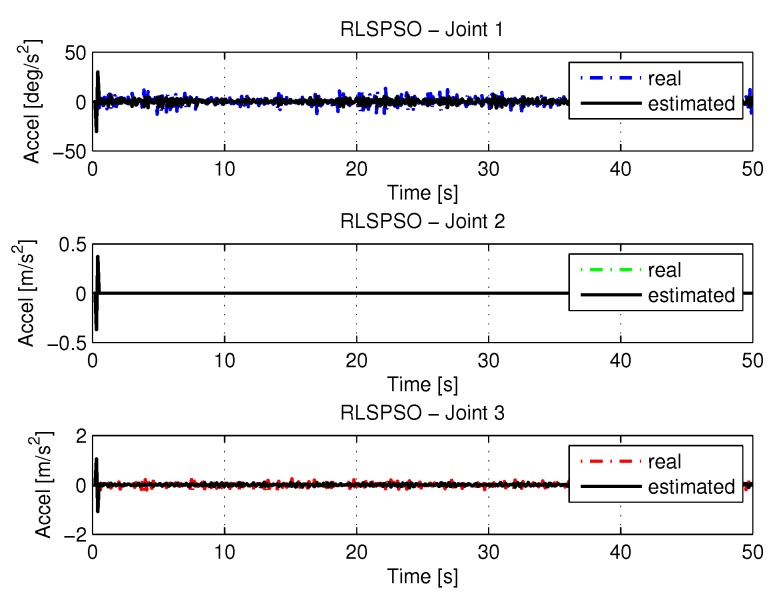
Accelerations of joints with RLSPSO with noise.

Figure 35 and Figure 36 shows the the speeds and accelerations of joints 1, 2, and 3 to perform the trajectories shown in Figure 33.

**Figure 37:**
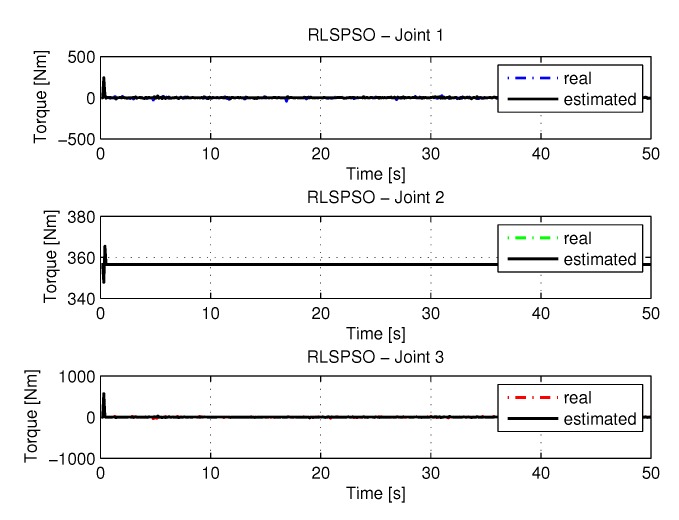
Torque of joints with RLSPSO with noise.

The torques obtained with the noisy inputs can be seen in Figure 37.

### 5.3. Comparison of Algorithms

A quantitative analysis of the each algorithm in the identification of the paths of each joint is given in Table 3 by the performance indexes: Multiple Correlation Coefficient, ($R^{2}$) and Computational Cost of each algorithms. Equation (29) presents $R^{2}$ given by,

(29) $$R^{2}=1-\frac{\sum_{i=1}^{n}{\left(y_{i}-{\hat{y}}_{i}\right)}^{2}}{\sum_{i=1}^{n}{\left(y_{i}-{\bar{y}}_{i}\right)}^{2}}$$

where $y_{i}$ are the observed data, $\bar{y}$ is mean of the observed data, and $\hat{y}$ data estimated by the model.

Can be observed from Table 3, the $R^{2}$ values of each joint are assumed values varies from 0 to 1, the closer to 1 means that the estimate is good.

Table 4 shows the results of the $R^{2}$ and computational cost of algorithms with noises.

From Table 3 can be observed that the index $R^{2}$ of the algorithm RLSPSO has a better result than the LS and RLS as well as a lower computational cost compared to the conventional RLS but was higher than that of the LS. For this application the proposed algorithm presented a better performance than the conventional RLS algorithm. The RLSPSO algorithm in this work is presented as a form of improvement of conventional RLS. Noise input methods achieved satisfactory results when compared to noisy methods.

Table 5 presents the complexity of the LS, RLS, and RLSPSO algorithms in terms of number of sums, multiplication, and divisions. It can be assumed that regressor vector has length M.

## 6. Discussion

Regarding the convergence of the RLSPSO algorithm: noise-free in the sixth iteration the algorithm converges for the best result and noise also begins to converge in the sixth iteration and fully converges in the forty-fifth iteration. Compared to classic RLS obtained an improvement in computational cost and overall result.

The main difficulty in the classical method is in weighting of covariance matrix that is empirically initialized. The metaheuristic pondered this matrix in a search space optimally so the covariance matrix was found faster and more efficiently than empirically or exhaustively searching. However this fact took into account the robustness of the RLSPSO algorithm because even being injected noise in the inputs managed to converge quickly and obtaining satisfactory results. The non-linearity of the system and the changes of operating points made identification difficult it is worth noting that the proposal may be valid as an alternative for nonlinear and variant systems.

Inadequate choice of covariance matrix may compromise method identification, so PSO was able to obtain a covariance matrix that could be robust enough to perform well even with data noise.

The speed and acceleration of a manipulator while performing a manipulation task depend on: grip stability, working environment, material shape, weight, material and stiffness of the object to be manipulated, type of grip or tool used. A cylindrical manipulator can be designed for high rigidity and load capacity and is suitable for transferring oversized materials, handling some parts or handling simple tools, not suitable for other tasks such as welding, assembling, grinding and usually work at low speeds [22,23]. For material handling tasks, the end-effector consists of a jaw of appropriate shape and size, determined by the object to be grasped. For machining and assembly tasks, the end-effector is a specialized tool or device, for example, a welding torch, a spray gun, a mill, a drill bit or a screwdriver [23].

For this paper, the data were collected at low speed because it is intended to use the manipulator for the displacement of high mass loads and lower speeds will be necessary because the material of the tool may slip, which also depends on the type of grip used, high speeds may occur as the material comes loose causing accidents. Another task that is intended to be used with the manipulator under study is the inspection where a camera will be used in place of the end-effector, to perform product quality inspection. The speeds we want to apply are in the range 0.1 to 1 m/s (linear speeds) and 5 to 50 deg/s (angular speeds) [23] for manipulation tasks. For inspection tasks the speed of the camera (which will be mounted on the end-effector) will be in the range of 0.10 to 0.30 m/s [35].

### More Method Results

More results of the identification of each method are presented here, where a more detailed comparison was made for a better visualization. Figure 38, Figure 39, Figure 40 and Figure 41 show the identifications of the methods noise-free noise and Figure 42, Figure 43, Figure 44 and Figure 45 with noise.

## 7. Conclusions

This work presents an alternative algorithm for calculating the inverse kinematics of robot manipulators based in RLS with PSO identification methods. Other methods were used, assessed and compared, namely LS and RLS. The results shown to be consistent and satisfactory in the identification of the inverse kinematics of the manipulator. Noises have also been added to the data to make estimates more difficult and to check their robustness when working with outlies. To show the efficiency of the algorithms, the $R^{2}$ of each algorithm, for each joint was calculated. The RLSPSO algorithm presented a better result than the conventional RLS both in $R^{2}$ and in computational cost. This algorithm is a form of improvement on the conventional RLS. This research also presented the kinematic and dynamic modelling of the manipulator. The dynamic model is important for the control of the manipulator. Also research is being performed on trajectory planning in a collision-free environment.

## Figures and Tables

**Figure 38:**
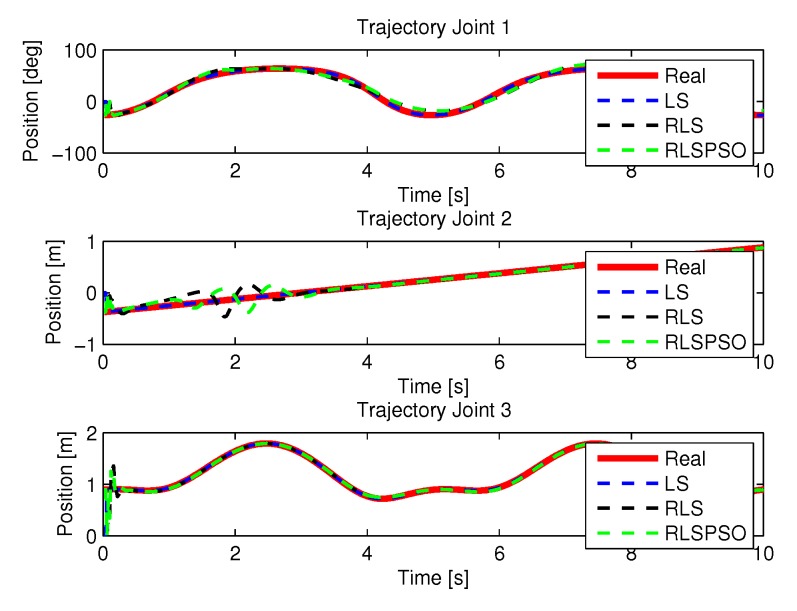
Trajectories identifications.

**Figure 39:**
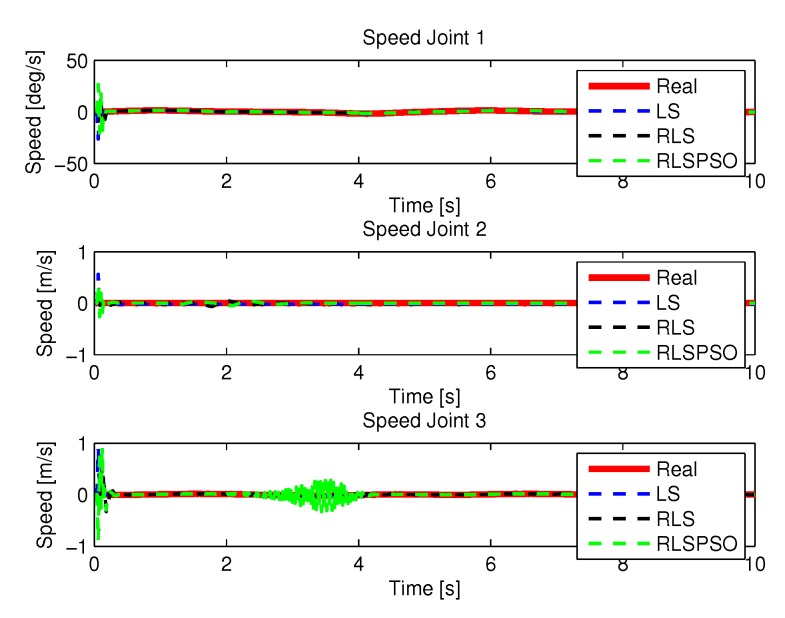
Speed identifications.

**Figure 40:**
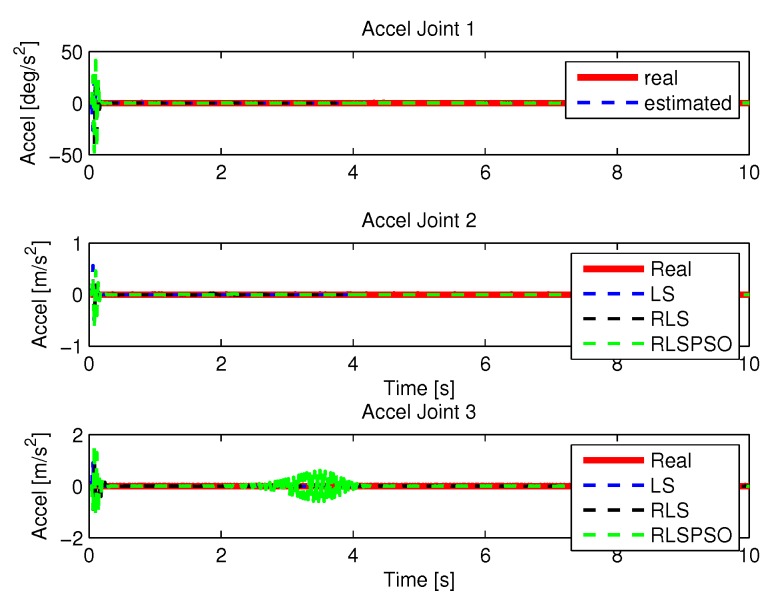
Acceleration identifications.

**Figure 41:**
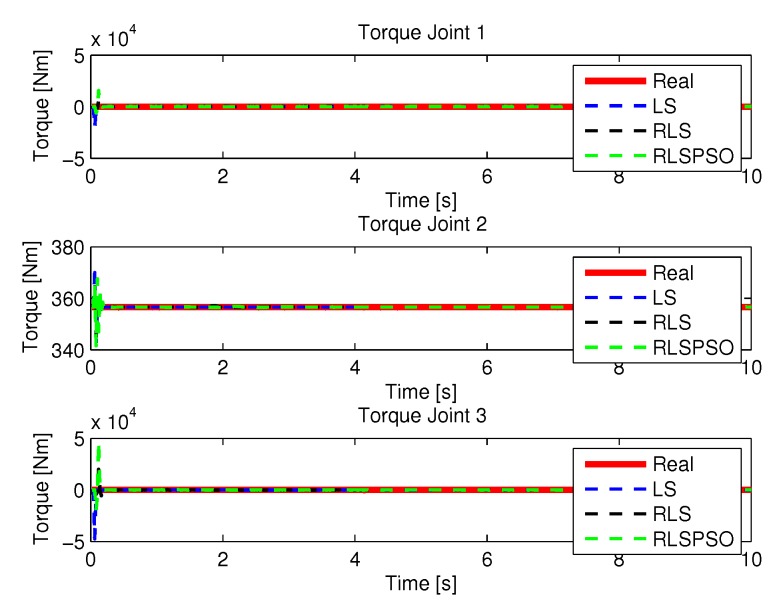
Torque identifications.

**Figure 42:**
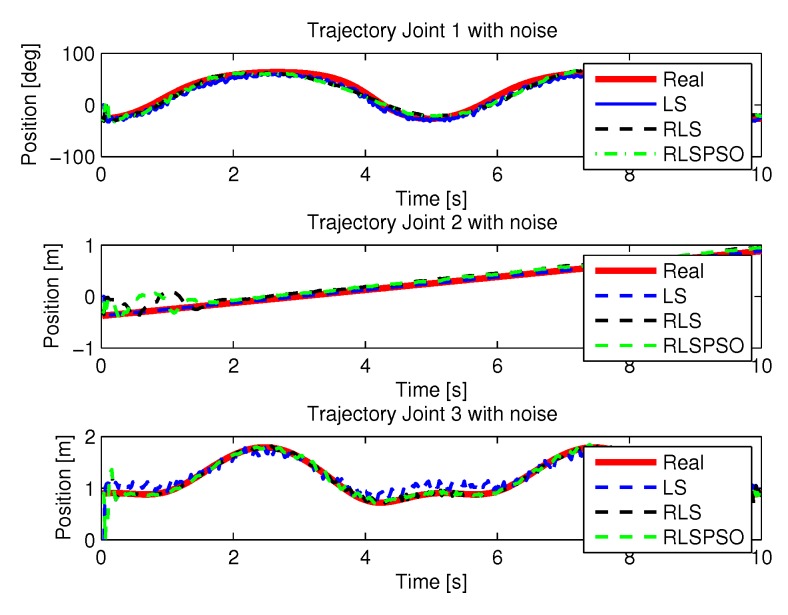
Trajectories with noise identifications.

**Figure 43:**
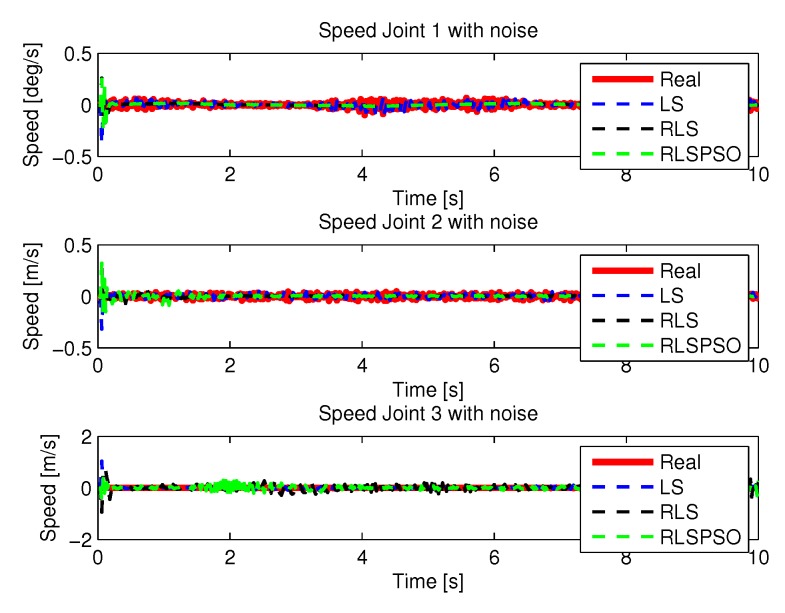
Speed with noise identifications.

**Figure 44:**
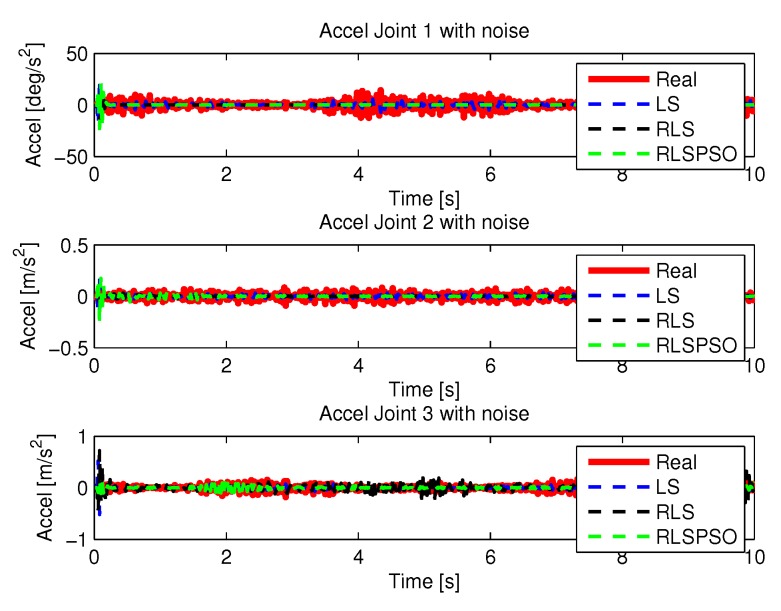
Acceleration with noise identifications.

**Figure 45:**
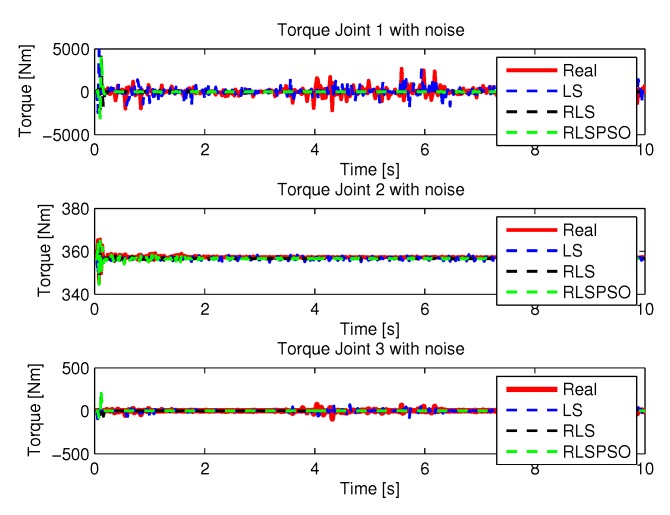
Torque with noise identifications.

**Table 1:** Values of masses (*m*) and lengths (*l*) of each link.

Link	*m* (kg)	*l* (m)
1 ($\theta_{1}$)	36.367405	0.050
2 ($d_{2}$)	12.632222	0.790
3 ($d_{3}$)	23.735183	0.900

**Table 2:** DH Parameters of an RPP manipulator.

Link	$a_{i}$	$\alpha_{i}$	$d_{i}$	$\theta_{i}$
1	0	0	0.245	$\theta_{1}$
2	0.11	−π/2	$d_{2}$	0
3	0	0	$d_{3}$	0

**Table 3:** Indexes $R^{2}$ (Joint 1, 2 and 3) and computational cost of algorithms.

Method	$R_{J_{1}}^{2}$	$R_{J_{2}}^{2}$	$R_{J_{3}}^{2}$	Comp. Cost (s)
LS	0.8873	0.7858	0.6829	2.565149
RLS	0.7946	0.7652	0.8408	65.039719
RLSPSO	0.8016	0.8017	0.8510	37.585912

**Table 4:** Indexes $R^{2}$ (Joint 1, 2 and 3) and computational cost of algorithms with noises.

Method	$R_{J_{1}}^{2}$	$R_{J_{2}}^{2}$	$R_{J_{3}}^{2}$	Comp. Cost (s)
LS	0.8129	0.7275	0.6129	2.851231
RLS	0.7321	0.7118	0.8012	73.989122
RLSPSO	0.7971	0.7912	0.8221	69.969319

**Table 5:** Complexity of Algorithms per input Sample.

Method	Additions	Multiplications	Divisions
LS	$2M^{2}+2M$	$4M^{2}+6M+1$	1
RLS	$3M^{2}+4M-1$	$6M^{2}+11M+1$	1
RLSPSO	$2M^{2}+3M-1$	$4M^{2}+8M+1$	1

## Appendix A. Dynamic Model of the Cylindrical Manipulator

The Lagrangian formulation based on the mechanical system is defined as

(A1) $$L\left(q,\dot{q}\right)=K\left(q,\dot{q}\right)-P\left(q\right)$$

For the cylindrical manipulator under study the kinetic and potential energy was calculated and then the Lagrangian based formulation was applied.

### Appendix A.1. Kinetic Energy

The total kinetic energy of the drive with drive for the three joints is given by [19]:

(A2) $$K=K_{1}+K_{2}+K_{3}$$

where

(A3) $$K_{1}=\frac{1}{2}\left[m_{1}v_{c1}^{2}+\omega_{1}^{T}I_{1}\omega_{1}\right],$$

(A4) $$K_{2}=\frac{1}{2}\left[m_{2}v_{c2}^{2}+\omega_{2}^{T}I_{2}\omega_{2}\right]$$

and

(A5) $$K_{3}=\frac{1}{2}\left[m_{3}v_{c3}^{2}+\omega_{3}^{T}I_{3}\omega_{3}\right]$$

From the Jacobian presented in (12) one can determine the speeds and the equations of the kinetic energy are

(A6) $$K_{1}=\frac{1}{2}\left[m_{1}{\left(-cos\theta_{1}d_{3}{\dot{\theta }}_{1}+sen\theta_{1}{\dot{d}}_{3}\right)}^{2}\right],$$

(A7) $$K_{2}=\frac{1}{2}\left[m_{2}{\left(-sen\theta_{1}d_{3}{\dot{\theta }}_{1}+cos\theta_{1}{\dot{d}}_{3}\right)}^{2}\right],$$

and

(A8) $$K_{3}=\frac{1}{2}\left[m_{3}{\dot{d}}_{2}^{2}+{\dot{\theta }}_{1}^{2}I_{3}\right]$$

After performing all the operations and some trigonometric transformations we have the equation that represents the total kinetic energy

(A9) $$\begin{array}{c} K=\frac{1}{2}[\left(m_{1}+m_{2}\right)\left(-2cos\theta_{1}sen\theta_{1}\right)\left(d_{3}{\dot{d}}_{3}{\dot{\theta }}_{1}\right)+\left(m_{1}cos^{2}\theta_{1}+m_{2}sen^{2}\theta_{1}\right)\left(d_{3}^{2}{\dot{\theta }}_{1}^{2}\right)+\\ \left(m_{1}sen^{2}\theta_{1}+m_{2}cos^{2}\theta_{1}\right)\left({\dot{d}}_{3}^{2}\right)+m_{3}{\dot{d}}_{2}^{2}+{\dot{\theta }}_{1}^{2}I_{3}] \end{array}$$

### Appendix A.2. Potential Energy

Starting from the definitions of the classical mechanics of reference point (zero of potential energy) the potential energy for each joint of the manipulator is

(A10) $$P_{1}=m_{1}gl_{1}sen\theta_{1}=0$$

because $l_{1}=a_{1}=0$,

(A11) $$P_{2}=m_{2}gd_{2}$$

and

(A12) $$P_{3}=m_{3}gd_{2}$$

As $P=P_{1}+P_{2}+P_{3}$, we have

(A13) $$P=gd_{2}\left(m_{2}+m_{3}\right)$$

### Appendix A.3. Lagrange Equation

The equations of motion of the system are given by

(A14) $$\frac{d}{dt}\left[\frac{\partial L}{\partial \dot{q}}\right]-\frac{\partial L}{\partial q}=\tau$$

where $\tau \in {ℜ}^{n}$ are the torques (forces) applied to the joints. Thus, considering the kinetic energy of the manipulator, the dynamic equation of the manipulator can be written in simplified form as

(A15) $$M\left(q\right)\ddot{q}+C\left(q,\dot{q}\right)\dot{q}+G\left(q\right)=\tau$$

where $C\in {ℜ}^{n}$ is the matrix that describes the centripetal and Coriolis forces, and $G=\frac{\partial g}{\partial q}\in {ℜ}^{n}$ is the gravity vector.

The effects of joint friction and external forces on the end-effector can be included in the dynamic model of the manipulator

(A16) $$M\left(q\right)\ddot{q}+C\left(q,\dot{q}\right)\dot{q}+F\left(q\right)q+G\left(q\right)=\tau -f_{ext}$$

On what $f_{ext}$ is the external force applied at the end-effector and $F\left(q\right)\in {ℜ}^{\times n}$ represents the effects of dynamic and static friction forces on the joints. This vector also represents disturbances and dynamics not modeled as gaps in couplings and mechanical transmissions.

Applying the Lagrange formulation (A1), the Lagrangian for the system will be

(A17) $$\begin{array}{c} L=\frac{1}{2}[\left(m_{1}+m_{2}\right)\left(-2cos\theta_{1}sen\theta_{1}\right)\left(d_{3}{\dot{d}}_{3}{\dot{\theta }}_{1}\right)+\left(m_{1}cos^{2}\theta_{1}+m_{2}sen^{2}\theta_{1}\right)\left(d_{3}^{2}{\dot{\theta }}_{1}^{2}\right)+\\ \left(m_{1}sen^{2}\theta_{1}+m_{2}cos^{2}\theta_{1}\right)\left({\dot{d}}_{3}^{2}\right)+m_{3}{\dot{d}}_{2}^{2}+{\dot{\theta }}_{1}^{2}I_{3}]-gd_{2}\left(m_{2}+m_{3}\right) \end{array}$$

The equation of the manipulator motion from the Lagrangian formulation is obtained by the partial derivatives of the Lagrangian Equation (A17). The following are the partial derivatives for the first manipulator joint

(A18) $$\frac{\partial L}{\partial {\dot{\theta }}_{1}}=\left(m_{1}+m_{2}\right)\left(-sen\theta_{1}cos\theta_{1}\right)\left(d_{3}{\dot{d}}_{3}\right)+\left(m_{1}cos\theta_{1}^{2}+m_{2}sen\theta_{1}^{2}\right)\left(d_{3}^{2}{\dot{\theta }}_{1}\right)+{\dot{\theta }}_{1}I_{3}$$

(A19) $$\frac{d}{dt}\frac{\partial L}{\partial {\dot{\theta }}_{1}}=\left(m_{1}+m_{2}\right)\left(cos\theta_{1}sen\theta_{1}\right)\left({\dot{d}}_{3}{\ddot{d}}_{3}\right)-2\left(2m_{1}sen\theta_{1}-2m_{2}cos\theta_{1}\right)\left({\dot{d}}_{3}{\ddot{\theta }}_{1}\right)+{\ddot{\theta }}_{1}I_{3}$$

(A20) $$\frac{\partial L}{\partial \theta_{1}}=\left(m_{1}+m_{2}\right)\left(sen\theta_{1}cos\theta_{1}\right)\left(d_{3}{\dot{d}}_{3}{\dot{\theta }}_{1}\right)-\left(m_{1}sen\theta_{1}-m_{2}cos\theta_{1}\right)\left({\dot{d}}_{3}{\dot{\theta }}_{1}^{2}\right)+\left(m_{1}cos\theta_{1}-m_{2}sen\theta_{1}\right)\left({\dot{d}}_{3}^{2}\right)$$

The first equation of motion describing the torque of joint 1 will be:

(A21) $$\begin{array}{c} \tau_{1}=-\left[\left(4m_{1}sen\theta_{1}-4m_{2}cos\theta_{1}\right)d_{3}+I_{3}\right]{\ddot{\theta }}_{1}\\ +\left[\left(m_{1}+m_{2}\right)\left(sen\theta_{1}cos\theta_{1}\right)d_{3}\right]{\ddot{d}}_{3}\\ +\left[\left(m_{1}sen\theta_{1}-m_{2}cos\theta_{1}\right)d_{3}\right]{\dot{\theta }}_{1}^{2}\\ -\left[m_{1}cos\theta_{1}+m_{2}sen\theta_{1}\right]{\dot{d}}_{3}^{2}\\ -\left[\left(m_{1}+m_{2}\right)\left(sen\theta_{1}cos\theta_{1}\right)d_{3}\right]{\dot{\theta }}_{1}{\dot{d}}_{3} \end{array}$$

In the same way, the partial derivatives for the second joint of the manipulator

(A22) $$\frac{\partial L}{\partial {\dot{d}}_{2}}=m_{3}{\dot{d}}_{2}$$

(A23) $$\frac{d}{dt}\frac{\partial L}{\partial {\dot{d}}_{2}}=m_{3}{\ddot{d}}_{2}$$

(A24) $$\frac{\partial L}{\partial d_{2}}=-g\left(m_{2}+m_{3}\right)$$

The equation of motion describing the torque of the joint 2 will be

(A25) $$\tau_{2}=m_{3}{\ddot{d}}_{2}+g\left(m_{2}+m_{3}\right)$$

Following the same idea, the partial derivatives for the third joint of the manipulator will be

(A26) $$\frac{\partial L}{\partial {\dot{d}}_{3}}=\left(m_{1}+m_{2}\right)\left(-sen\theta_{1}cos\theta_{1}d_{3}{\dot{\theta }}_{1}\right)+2\left(m_{1}cos\theta_{1}-2m_{2}sen\theta_{1}\right){\dot{d}}_{3}$$

(A27) $$\frac{d}{dt}\frac{\partial L}{\partial {\dot{d}}_{3}}=\left[m_{1}sen\theta_{1}cos\theta_{1}\right]{\ddot{\theta }}_{1}-\left[2\left(m_{1}sen\theta_{1}+m_{2}cos\theta_{1}\right)\right]{\ddot{d}}_{3}+\left[m_{2}sen\theta_{1}cos\theta_{1}\right]{\dot{d}}_{3}$$

(A28) $$\frac{\partial L}{\partial d_{3}}=\left(m_{1}+m_{2}\right)\left(sen\theta_{1}cos\theta_{1}\right)\left({\dot{d}}_{3}{\dot{\theta }}_{1}\right)-\left(2m_{1}sen\theta_{1}-2m_{2}cos\theta_{1}\right)\left(d_{3}{\dot{\theta }}_{1}^{2}\right)$$

The equation of motion describing the torque of the joint 3 will be

(A29) $$\begin{array}{c} \tau_{3}=\left[m_{1}sen\theta_{1}cos\theta_{1}\right]{\ddot{\theta }}_{1}\\ -\left[2\left(m_{1}sen\theta_{1}+m_{2}cos\theta_{1}\right)\right]{\ddot{d}}_{3}\\ +\left[2d_{3}\left(m_{1}sen\theta_{1}-m_{2}cos\theta_{1}\right)\right]{\dot{\theta }}_{1}^{2}\\ -\left[\left(m_{1}+m_{2}\right)\left(sen\theta_{1}cos\theta_{1}\right)\right]{\dot{\theta }}_{1}{\dot{d}}_{3} \end{array}$$

### Appendix A.4. Dynamics of the Matrix form Manipulator

Writing Equations (A21), (A25) and (A29) in the standard matrix form as shown in (A15) we have

(A30) $$\begin{array}{c} \left[\begin{array}{c} \tau_{1}\\ \tau_{2}\\ \tau_{3} \end{array}\right]=\left[\begin{array}{ccc} \left(4m_{1}sen\theta_{1}-4m_{2}cos\theta_{1}\right)d_{3}+I_{3} & 0 & \left(m_{1}+m_{2}\right)\left(sen\theta_{1}cos\theta_{1}\right)d_{3}\\ 0 & m_{3} & 0\\ m_{1}sen\theta_{1}cos\theta_{1} & 0 & 2(m_{1}sen\theta_{1}+m_{2}cos\theta_{1}) \end{array}\right]\times \left[\begin{array}{c} {\ddot{\theta }}_{1}\\ {\ddot{d}}_{2}\\ {\ddot{d}}_{3} \end{array}\right]\\ +\left[\begin{array}{ccc} \left(m_{1}sen\theta_{1}-m_{2}cos\theta_{1}\right)d_{3} & 0 & -m_{1}cos\theta_{1}+m_{2}sen\theta_{1}\\ 0 & 0 & 0\\ 2d_{3}\left(m_{1}sen\theta_{1}-m_{2}cos\theta_{1}\right) & 0 & 0 \end{array}\right]\times \left[\begin{array}{c} {\dot{\theta }}_{1}^{2}\\ {\dot{d}}_{2}^{2}\\ {\dot{d}}_{3}^{2} \end{array}\right]\\ +\left[\begin{array}{ccc} 0 & -\left(m_{1}+m_{2}\right)\left(sen\theta_{1}cos\theta_{1}\right)d_{3} & 0\\ 0 & 0 & 0\\ 0 & -\left(m_{1}+m_{2}\right)\left(sen\theta_{1}cos\theta_{1}\right) & 0 \end{array}\right]\times \left[\begin{array}{c} {\dot{\theta }}_{1}{\dot{d}}_{2}\\ {\dot{\theta }}_{1}{\dot{d}}_{3}\\ {\dot{d}}_{2}{\dot{d}}_{3} \end{array}\right]\\ +\left[\begin{array}{c} 0\\ g(m_{2}+m_{3})\\ 0 \end{array}\right] \end{array}$$

In Equation (A30), the terms ${\ddot{\theta }}_{1},\;{\ddot{d}}_{2},\;{\ddot{d}}_{3}$ are related to the angular accelerations of the links, the terms ${\dot{\theta }}_{1}^{2},\;{\dot{d}}_{2}^{2},\;{\dot{d}}_{3}^{2}$ are centripetal accelerations, and the terms ${\dot{\theta }}_{1}{\dot{d}}_{2},\;{\dot{\theta }}_{1}{\dot{d}}_{3},\;{\dot{d}}_{2}{\dot{d}}_{3}$ are the Coriolis accelerations.
